# Uric Acid Functions as an Endogenous Modulator of Microglial Function and Amyloid Clearance in Alzheimer's Disease

**DOI:** 10.1002/advs.202510270

**Published:** 2025-10-06

**Authors:** De Xie, Qiuyang Zheng, Jiaming Lv, Qian Zhang, Zhiwei Cui, Shuai Huang, Wei Yu, Binyang Chen, Wanling Que, Shanpan Fu, Yuemei Xi, Jiayu Chen, Xueling Ye, Shuyi Chen, Hairong Zhao, Tetsuya Yamamoto, Hidenori Koyama, Xin Wang, Jidong Cheng

**Affiliations:** ^1^ Department of Internal Medicine, Xiang'an Hospital of Xiamen University, School of Medicine Xiamen University Xiamen Fujian 361102 China; ^2^ Xiamen Key Laboratory of Translational Medicine for Nucleic Acid Metabolism and Regulation Xiamen Fujian 361102 China; ^3^ State Key Laboratory of Cellular Stress Biology, Fujian Provincial Key Laboratory of Neurodegenerative Disease and Aging Research, Institute of Neuroscience, School of Medicine, Shenzhen Research Institute Xiamen University Xiamen Fujian 361102 China; ^4^ Department of Geriatrics Shaanxi Provincial People's Hospital Xi'an Shaanxi 710061 China; ^5^ The First Affiliated Hospital of Xi'an Jiaotong University Xi'an Shaanxi 710061 China; ^6^ Department of Health Evaluation Center Osaka Gyoumeikan Hospital Osaka 554‐0012 Japan; ^7^ Department of Diabetes, Endocrinology and Clinical Immunology Hyogo College of Medicine Nishinomiya Hyogo 663‐8501 Japan

**Keywords:** Alzheimer's disease, amyloid‐β, microglia, uric acid

## Abstract

Epidemiological studies have linked uric acid (UA), the end product of purine metabolism in humans, with reduced Alzheimer's disease (AD) risk. Decreased serum UA levels are observed in AD patients versus age‐matched controls, while upstream purine metabolites remained unchanged. In 5×FAD mice, two months of UA supplementation improved cognitive function and reduced amyloid plaque burden. Mechanistically, UA enhances microglial amyloid‐β (Aβ) phagocytosis and induces transcriptional reprogramming in AD mouse microglia, characterized by upregulated phagocytic pathways and attenuated inflammatory responses. UA treatment restored the recycling of Aβ receptors CD36 and TREM2 in microglia, enhanced lysosomal biogenesis, and facilitated Aβ degradation. These findings identify UA as a critical endogenous modulator of microglial Aβ processing and suggest exploring UA supplementation as a therapeutic strategy for AD.

## Graphical abstract

Higher serum UA levels are associated with reduced AD risk in epidemiological studies. UA enhances microglial phagocytic capacity primarily by restoring CD36 and TREM2 receptor recycling that is impaired by Aβ exposure. Therapeutically, UA supplementation improves cognitive function and reduces neuropathology in AD mouse model by enhancing microglial Aβ clearance.

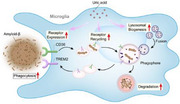

## 1 Introduction

Alzheimer's disease (AD), a progressive neurodegenerative disease, is the predominant cause of age‐related dementia,^[^ ^1^ ^]^ affecting ≈6.7 million individuals aged 65 and older in the United States alone.^[^ ^2^ ^]^ The pathological hallmarks of AD comprise two distinct protein aggregates: extracellular amyloid plaques and intraneuronal neurofibrillary tangles.^[^ ^3^ ^]^ The amyloid hypothesis, supported by extensive pathological, epidemiological, and genetic evidence, posits amyloid‐β (Aβ) accumulation as a central driver of AD pathogenesis.^[^ ^4^, ^5^ ^]^ Through the amyloidogenic pathway, sequential proteolytic processing of amyloid precursor protein (APP) by β‐ and γ‐secretase generates Aβ peptides.^[^ ^6^ ^]^ These peptides subsequently oligomerize to form diffusible, neurotoxic species, representing early and critical events in AD molecular pathology.^[^ ^7^ ^]^ These Aβ oligomers trigger a cascade of pathological events, including mitochondrial dysfunction, synaptic deterioration, memory impairment, and progressive neurodegeneration.^[^ ^8^, ^9^ ^]^

Uric acid (UA), the end product of purine metabolism in humans, is ubiquitously distributed in intracellular and extracellular fluids.^[^ ^10^ ^]^ Elevated physiological UA levels in humans confer evolutionary advantages, including enhanced antioxidant capacity,^[^ ^11^, ^12^, ^13^ ^]^ blood pressure regulation,^[^ ^14^, ^15^ ^]^ and anti‐aging effects.^[^ ^16^, ^17^ ^]^ UA also exhibits neuroprotective properties in various neurological disorders, including Parkinson's disease,^[^ ^18^ ^]^ ischemic stroke,^[^ ^17^, ^19^ ^]^ and multiple sclerosis.^[^ ^20^, ^21^ ^]^ Epidemiological studies have demonstrated an inverse correlation between serum UA (SUA) and AD risk.^[^ ^22^, ^23^ ^]^ Moreover, elevated baseline SUA levels are associated with improved AD‐related cerebral hypometabolism and cognitive performance.^[^ ^24^ ^]^ A comprehensive database analysis demonstrated that higher SUA levels confer protection against cognitive decline, both independently and synergistically with cerebrospinal fluid (CSF) AD biomarkers, including Aβ_1–42_ and tau.^[^ ^25^ ^]^ However, conflicting findings regarding UA's role in AD necessitate further mechanistic investigation.

Microglia, the brain's resident immune cells, serve as long‐lived phagocytes in the central nervous system,^[^ ^26^ ^]^ orchestrating the recognition and scavenging of dying neurons, pathogens, cellular debris, and aberrant protein aggregates.^[^ ^27^ ^]^ In AD pathology, microglia play a pivotal role through their capacity to recognize, phagocytose, and degrade Aβ,^[^ ^28^, ^29^ ^]^ as evidenced by their localization around amyloid plaques.^[^ ^30^ ^]^ However, senescence‐associated microglial dysfunction, characterized by compromised phagocytic capacity and chronic inflammatory activation, contributes to excessive Aβ deposition.^[^ ^31^, ^32^ ^]^ Given that Aβ deposition reflects an imbalance between its production and clearance, restoration of microglial functionality emerges as a compelling therapeutic strategy for AD intervention.

In this study, we investigated the impact of UA administration on amyloid pathology and cognitive deficits in 5×FAD model mice, with particular focus on microglial functions related to Aβ phagocytosis and degradation. Our results demonstrate that UA restored impaired CD36 and TREM2 recycling in microglia, enhanced Aβ and dextran phagocytosis, and promoted lysosomal degradation. These findings suggest that UA acts as a key endogenous regulator of microglial function, and that UA supplementation may represent a promising therapeutic strategy for AD intervention.

## 2 Results

### 2.1 Reduced UA Levels are Associated with Elevated AD Risk

**Figure 1:**
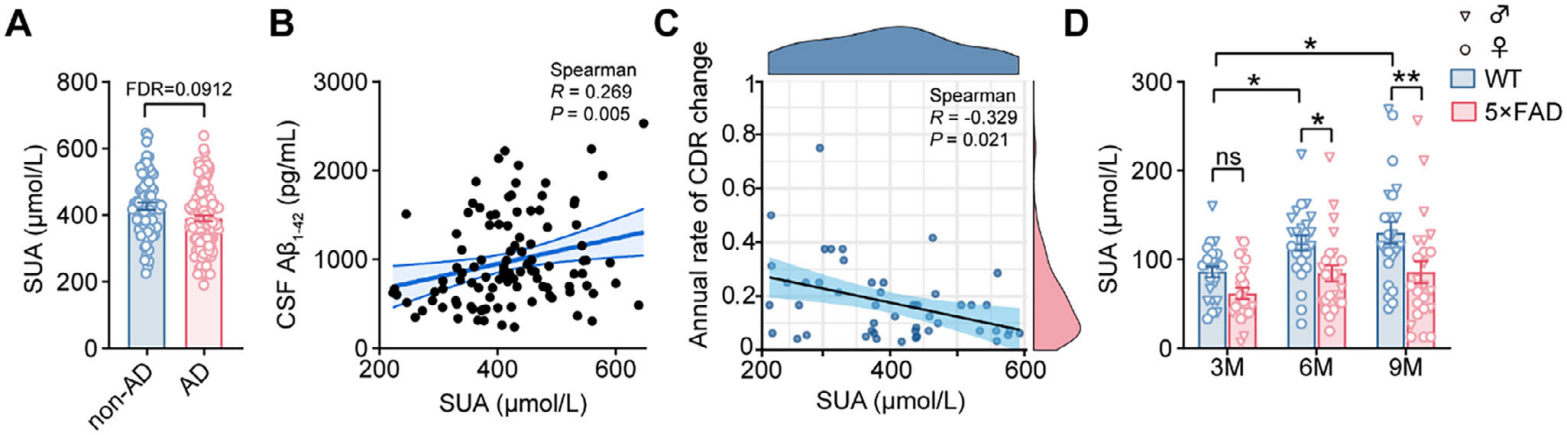
Decreased serum UA levels in AD patients and 5×FAD mice. A) Quantitative analysis of serum UA (SUA) levels in control subjects (non‐AD; n = 76) and AD patients (n = 124). Data were obtained from the ADNI database. B) Correlation analysis between baseline SUA and CSF Aβ_1‐42_ levels in non‐AD (n = 41) and AD subjects (n = 64). C) Correlation analysis between baseline SUA levels and annualized changes in Clinical Dementia Rating (CDR) scores among subjects with longitudinal follow‐up data (n = 49, subset from cohort in (A)). D) Age‐dependent comparison of SUA levels between WT and 5×FAD mice at 3, 6, and 9 months of age (n = 24 per genotype; triangles and circles denote male and female, respectively). Data are presented as mean ± SEM. *P* values in (A) were determined by a two‐tailed unpaired Student's *t*‐test and adjusted using the Benjamini–Hochberg false discovery rate (FDR, *q* < 0.10); *P* values in (B and C) were determined by Spearman's correlation analysis; *P* values in (D) were determined by two‐way ANOVA followed by Sidak's multiple comparisons test. ns, not significant. ^*^ *P* < 0.05 (for D); ^**^ *P* < 0.01 (for D).

The biochemical data of purine metabolites and related amino acids in human plasma were collected from the Alzheimer's Disease Neuroimaging Initiative (ADNI) database. Baseline demographic and clinical characteristics of healthy controls (non‐AD) and AD patients are summarized in Table S1 (Supporting Information). The study cohort comprised non‐AD (n = 76; 38%) and AD groups (n = 124; 62%), matched for age, sex, educational background, *APOE4* status, cardiovascular risk factors, and renal function. Clinical assessment revealed that AD patients exhibited a significantly lower Mini‐Mental State Examination (MMSE) score and reduced CSF Aβ_1‐42_ levels compared to non‐AD controls. Notably, among all baseline metabolites assessed, only SUA levels were markedly reduced in AD patients (390.9 ± 8.8 µmol L^−1^) compared to controls (426.7 ± 10.7 µmol L^−1^) (**Figure** **1**A; Figure S1A–G, Supporting Information). The levels of hypoxanthine and xanthine, direct precursors of UA in the purine metabolic pathway, showed no significant differences between AD and non‐AD groups (Figure S1E,F, Supporting Information). Baseline SUA levels were positively correlated with CSF Aβ_1‐42_ (Spearman R = 0.269, *P* = 0.005), but not with CSF Aβ_1‐40_ (*P* = 0.843) (Figure 1B; Figure S1H, Supporting Information). Furthermore, longitudinal analysis revealed that higher baseline SUA levels were associated with slower progression of cognitive decline, as measured by Clinical Dementia Rating (CDR) scores (Figure 1C). After adjusting for key covariates in model 2, SUA remained an independent predictor of CDR progression (β = −0.203 per 1 mg dL^−1^ increase in SUA; 95% CI: −0.053–−0.011; Table S2 (Supporting Information). Consistent with the human data, SUA levels were significantly lower in 5×FAD mice compared with wild‐type (WT) mice at both 6 and 9 months of age (Figure 1D). These findings corroborate previous studies,^[^ ^33^, ^34^ ^]^ and suggest a potential regulatory role for UA in dementia.

### 2.2 UA Restores Cognitive Dysfunction in 5×FAD Mice

In vivo microdialysis was performed to measure dynamic UA concentrations in brain interstitial fluid (ISF) upon intraperitoneal administration (Figure S2A–C, Supporting Information). After a single intraperitoneal injection of UA (200 mg kg^−1^), SUA level peaked at 15 min and declined rapidly within 2 h in both WT and 5×FAD mice (Figure S2D, Supporting Information). This transient increase followed by rapid decline was attributed to the rapid renal excretion of UA and the retention of uricase (urate oxidase) in mice.^[^ ^35^ ^]^ Concomitantly, ISF UA levels increased significantly at 1 h post‐injection (168.6 ± 31.3 µmol L^−1^ in WT vs 115.5 ± 16.9 µmol L^−1^ in 5×FAD mice) and remained elevated over time (Figure S2E, Supporting Information). Lower and prolonged UA levels in the dialysate suggested that peripheral changes influence the ISF UA dynamics. Similar dynamic changes were observed in both serum and ISF across WT and 5×FAD mice following UA treatment.

**Figure 2:**
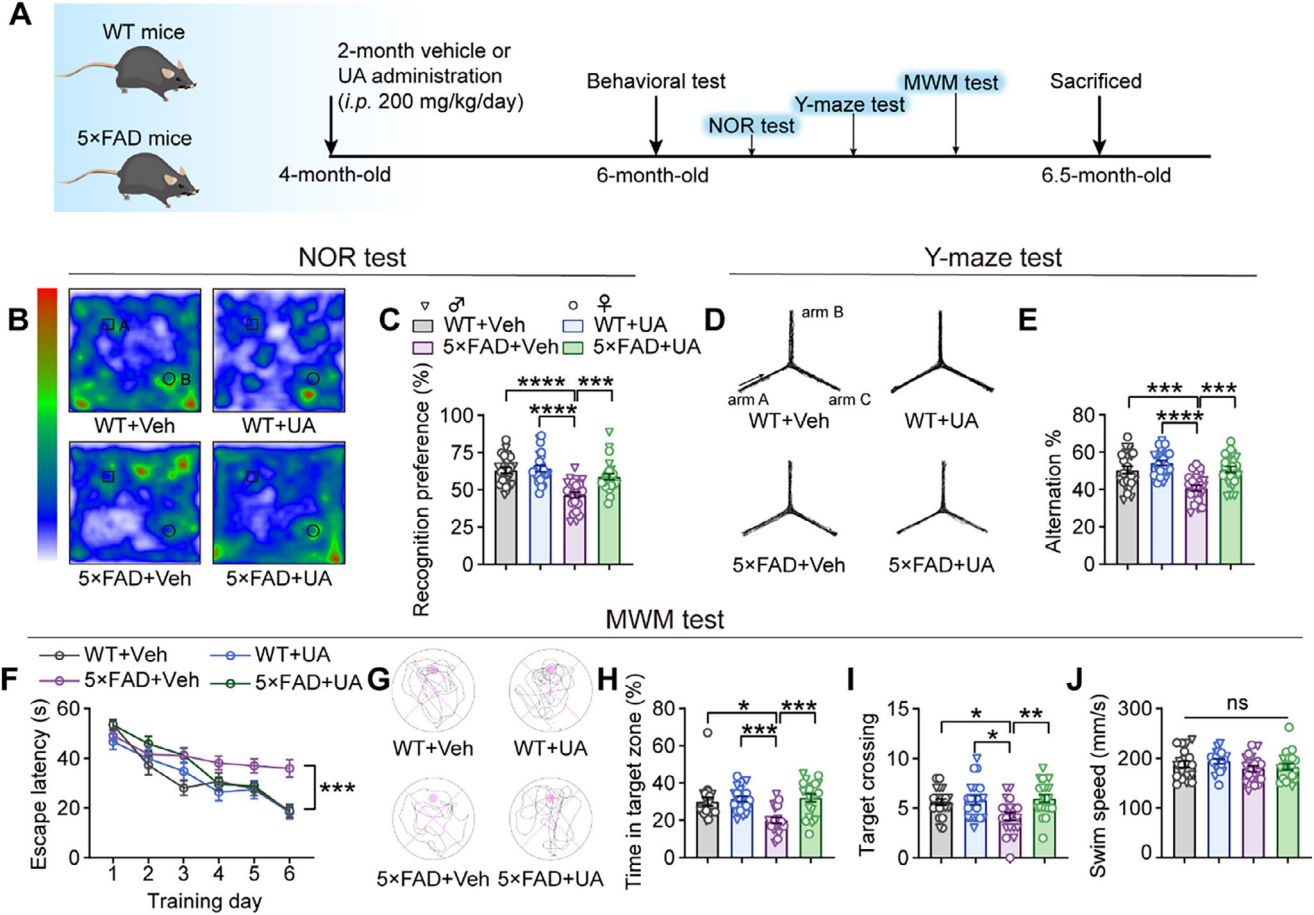
UA administration ameliorates cognitive deficits in 5×FAD mice. A) Four‐month‐old 5×FAD mice and their wild‐type (WT) littermates were intraperitoneal (*i.p*.) injection with vehicle (Veh) or UA (200 mg/kg/day) for 2 months and subjected to behavioral assessments, including novel object recognition (NOR), Y‐maze test, and Morris water maze (MWM), followed by comprehensive pathological analysis. The experimental groups consisted of: WT+Veh, WT+UA, 5×FAD+Veh, and 5×FAD+UA (n = 20–24 mice per group; triangles: males, circles: females). B) Representative heat maps of exploratory behavior (object A: familiar object; object B: novel object) and C) quantification of novel object preference in the NOR test. D) Representative locomotion trajectories and E) quantification of spontaneous alternation behavior in the Y‐maze test. F) Escape latency during the MWM training phase. G) Representative swim paths during the probe test on day 7. H) Time spent in the target quadrant, I) target crossing number, and J) mean swimming speed during the MWM probe test. Data are presented as mean ± SEM. *P* values were determined by one‐way ANOVA followed by Tukey's *post hoc* analysis in (C, E, I, and J), two‐way ANOVA followed by Sidak's multiple comparisons test in (F), and Kruskal–Wallis test followed by Dunn's *post hoc* analysis in (H). ns, not significant; ^*^ *P* < 0.05; ^**^ *P* < 0.01; ^***^ *P* < 0.001; ^****^ *P* < 0.0001.

To assess the therapeutic potential of UA, 4‐month‐old WT and 5×FAD mice received daily intraperitoneal injections of UA (200 mg kg^−1^) or vehicle (Veh) for 2 months. The dosage was selected to address the UA deficiency in AD patients, considering intraperitoneal bioavailability and the presence of uricase in mice. Consistent with previous pharmacological studies,^[^ ^36^ ^]^ UA administration at this dose did not induce hyperuricemia‐associated organ damage, inflammatory, or metabolic dysfunction, as evidenced by histological examination of vital organs and serum biochemistry analyses after 2‐week and 2‐month supplementation (Figure S3A–S, Supporting Information). After treatment, a series of cognitive behavior tests were performed to evaluate UA's therapeutic effects in the 5×FAD murine AD model (**Figure** **2**A). In the novel object recognition (NOR) test, 5×FAD+vehicle mice displayed no preference between the novel and familiar objects, indicating impaired recognition memory. In contrast, both WT groups and 5×FAD+UA mice spent significantly more time exploring the novel object, demonstrating improved recognition memory in the UA‐treated 5×FAD mice (Figure 2B,C). Similarly, the Y‐maze test revealed a significant increase in spontaneous alternation behavior, indicative of improved working memory, in 5×FAD mice treated with UA compared to vehicle‐treated 5×FAD mice (Figure 2D,E). In the Morris water maze (MWM) test, UA‐treated 5×FAD mice exhibited reduced escape latency during training sessions (Figure 2F). On the probe trial, UA‐treated 5×FAD mice spent significantly more time in the target quadrant and showed a clear preference for the target platform location, indicating enhanced spatial memory (Figure 2G–I). Importantly, UA‐treatment did not affect swimming speed, ruling out motor impairment as a confounding factor (Figure 2J). Additionally, UA treatment had no influence on cognitive performance in WT mice across all behavioral tests. Collectively, these results demonstrate that UA treatment significantly ameliorated spatial learning and memory deficits in the 5×FAD mouse model.

### 2.3 UA Reduces Amyloid Plaque Deposition in 5×FAD Mice

**Figure 3:**
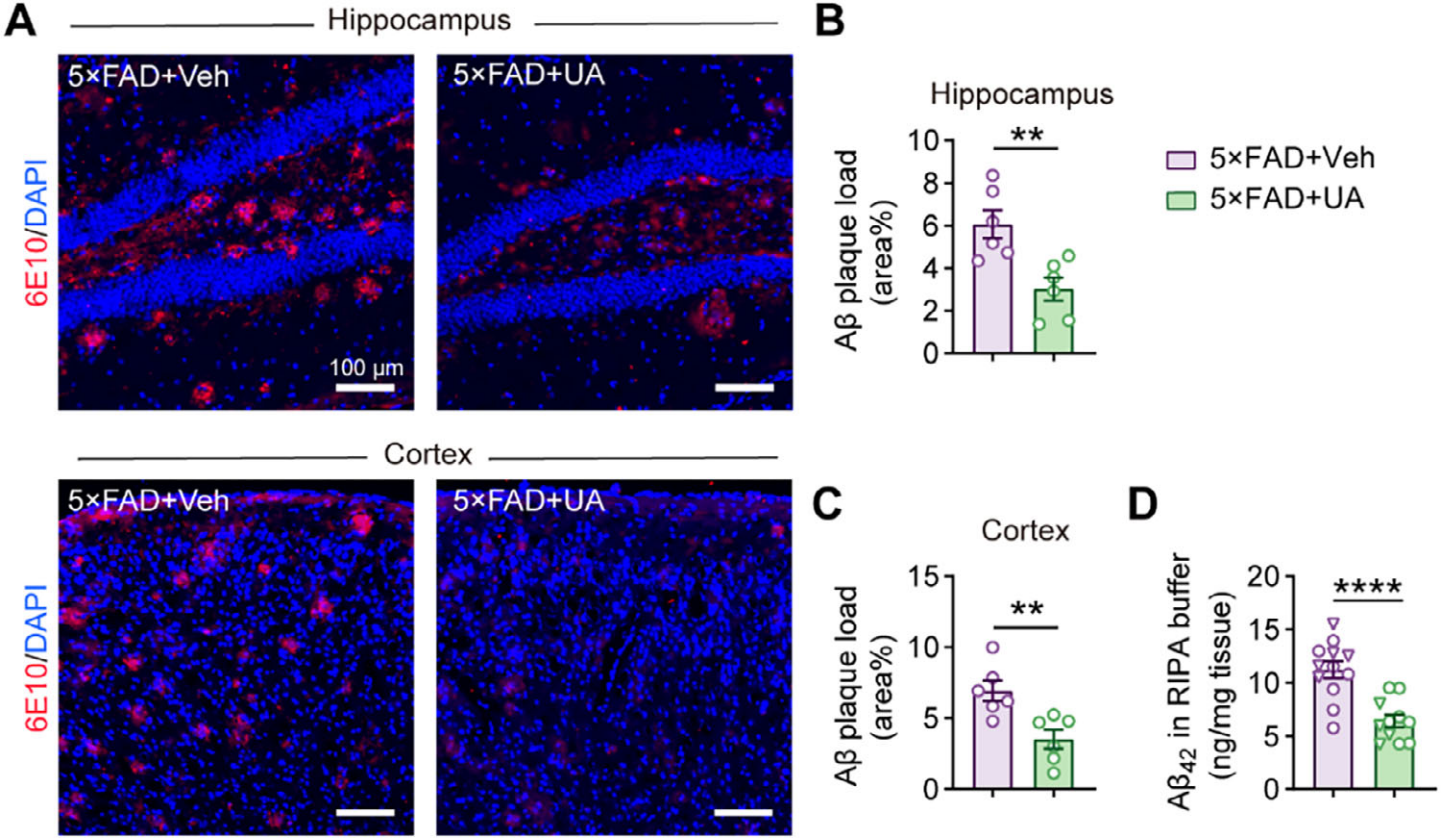
UA reduces amyloid plaque deposition in 5×FAD mice. Four‐month‐old 5×FAD mice received either UA or vehicle treatment for 2 months to evaluate amyloid deposition load in the brain. A) Immunofluorescence staining of amyloid plaques (6E10, red; DAPI, blue) in hippocampal and cortical sections. Scale bar, 100 µm. B,C) Quantification of amyloid plaques in (A) (n = 6 mice, 18 fields per group for analysis). D) Quantification of soluble Aβ_42_ in RIPA buffer extracted from cerebral cortices. n (5×FAD+Veh) = 12 mice (5 male, 7 female); n (5×FAD+UA) = 11 mice (4 male, 7 female). Triangles: males; circles: females. Data are presented as mean ± SEM. *P* values were determined by a two‐tailed unpaired Student's *t*‐test in (B–D). ^**^ *P* < 0.01; ^****^ *P* < 0.0001.

Consistent with the age‐dependent Aβ pathology in 5×FAD mice,^[^ ^37^ ^]^ UA treatment significantly reduced amyloid plaque deposition in both hippocampi and cortices of 5×FAD mice (**Figure** **3**A–C). No evident plaque deposition was observed in age‐matched WT mice treated with vehicle or UA (Figure S4A, Supporting Information). Furthermore, UA treatment reduced RIPA‐soluble Aβ_42_ levels in the cortices of 5×FAD mice (Figure 3D). UA supplementation increased antioxidant enzymes GPX4 and SOD2 and reduced the lipid peroxidation marker 4‐HNE in the hippocampi of 5×FAD mice (Figure S4B,C, Supporting Information), demonstrating its antioxidant effects.

### 2.4 UA Promotes Microglial Phagocytosis of Amyloid Plaques

**Figure 4:**
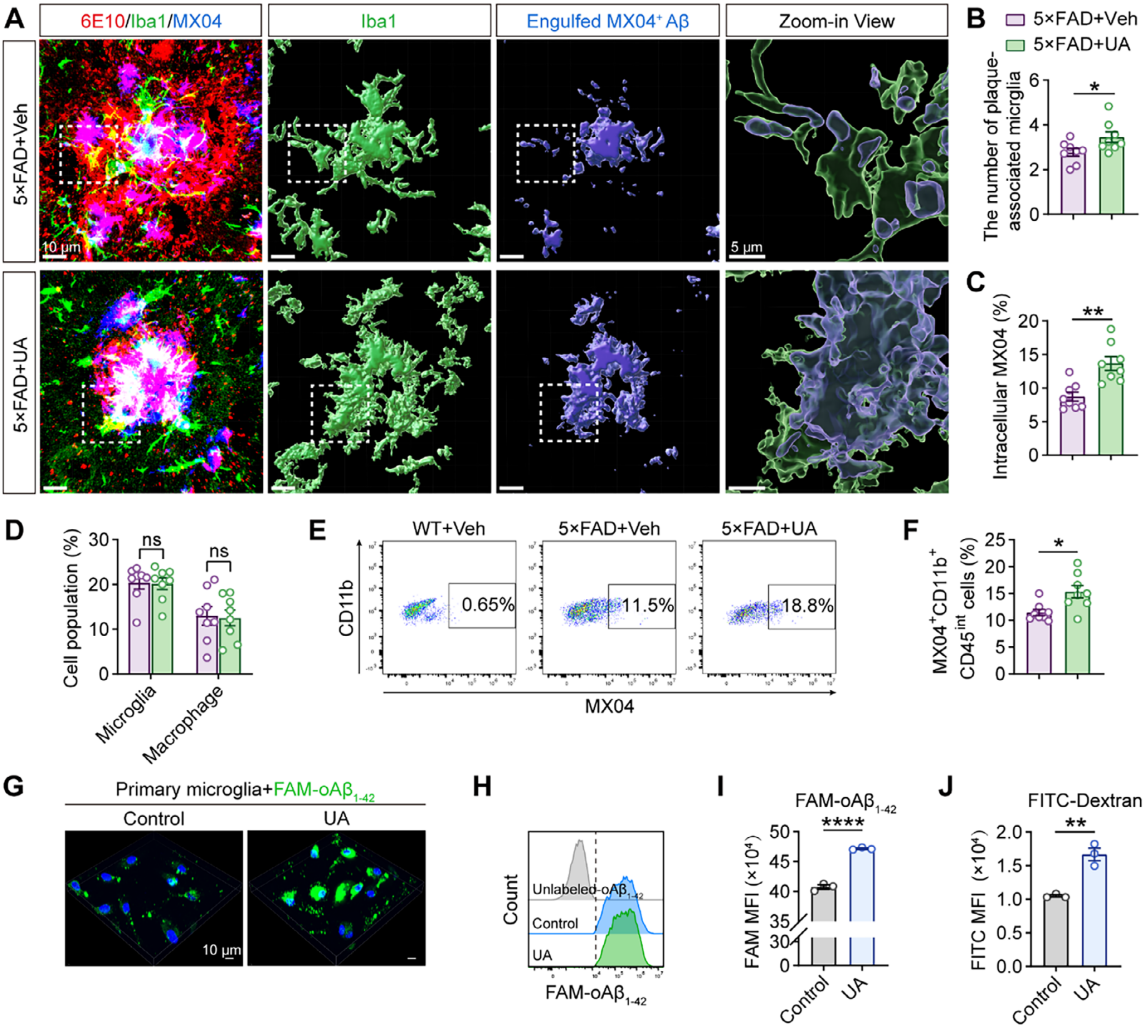
UA treatment enhances microglial phagocytosis of Aβ. A) Representative Imaris‐based 3D reconstruction of amyloid plaques (MX04, blue; 6E10, red) and microglia (Iba1, green). Scale bar, 10 µm; 5 µm in zoom‐in view. B) Quantification of the number of plaque‐associated microglia (n = 8 mice per group, 43 plaques for 5×FAD+Veh and 44 plaques for 5×FAD+UA). C) Quantification of the MX04 internalization by microglia. n = 53–55 plaques from 10 volumetric images of 8 mice per group. D) Flow cytometric quantification of microglial and macrophage populations in 5×FAD+Veh and 5×FAD+UA mice (n = 8 mice per group). E) Representative flow cytometry plots and F) quantification of MX04^+^CD11b^+^CD45^int^ hippocampal microglia from WT+Veh, 5×FAD+Veh, and 5×FAD+UA mice (n = 8 mice per group). G) Representative confocal z‐stack images and H) flow cytometry histograms of primary microglia exposed to FAM‐oAβ_1‐42_ (1 µm, 3 h) following treatment with vehicle or UA (100 µm, 12 h). I) Quantification of mean fluorescence intensity (MFI) from (H) (n = 3 independent experiments). J) Flow cytometry analysis of endocytic capacity using FITC‐conjugated dextran (1 µg mL^−1^, 3 h; n = 3 independent experiments). Data are presented as mean ± SEM. *P* values were determined by two‐tailed unpaired Student's *t*‐test in (B, C, D macrophage, F, I, and J), and two‐tailed Mann–Whitney test in (D microglia). ns, not significant; ^*^ *P* < 0.05; ^**^ *P* < 0.01; ^****^ *P* < 0.0001.

Given the crucial role of microglia as primary phagocytic cells in the brain,^[^ ^38^ ^]^ we investigated whether reduced amyloid plaque deposition reflected enhanced microglial function. Using immunofluorescence staining with microglial and amyloid plaque markers (6E10 and Methoxy‐X04 [MX04]; **Figure** **4**A), we observed increased plaque‐associated microglia surrounding amyloid plaques in UA‐treated 5×FAD mice (Figure 4B). Moreover, 3D reconstructions of Iba1 and MX04 confirmed significantly enhanced microglial phagocytosis of amyloid plaques following UA treatment (Figure 4C). To validate these findings in vivo, we isolated microglia from WT and 5×FAD mice for flow cytometric analysis following MX04 administration (Figure S5A, Supporting Information). While UA treatment did not alter the number of microglia (CD11b^+^CD45^int^) or macrophages (CD11b^+^CD45^high^; Figure 4D), the proportion of MX04^+^ microglia increased significantly in 5×FAD+UA mice (Figure 4E,F), indicating enhanced microglial phagocytic capacity following UA administration.

We further validated the effects of UA on Aβ uptake in primary microglia cultures. Following 12‐h UA treatment, we quantified phagocytosis of FAM‐labeled oligomeric Aβ_1‐42_ (FAM‐oAβ_1‐42_) and unlabeled oAβ_1‐42_ using immunofluorescence and flow cytometry. UA treatment (100 µm) markedly enhanced microglial Aβ phagocytosis (Figure 4G–I; Figure S5B–D, Supporting Information), an effect blocked by probenecid (Prob), a UA channel inhibitor (Figure S5E,F, Supporting Information).^[^ ^39^ ^]^ UA also facilitated the internalization of dextran (Figure 4J), suggesting broadly improved microglial phagocytic capacity. To investigate potential mechanisms, we examined the effect of N‐acetyl‐L‐cysteine (NAC; a ROS scavenger) and UA‐related purine metabolites. While NAC alone showed no effect, combined treatment with UA and NAC enhanced FAM‐oAβ_1‐42_ uptake by microglia (Figure S5G,H, Supporting Information). In contrast, UA metabolites (hypoxanthine, xanthine, guanine, and allantoin) attenuated Aβ uptake (Figure S5G,H, Supporting Information), suggesting UA‐specific regulation of microglial phagocytosis.

Given the role of astrocytes in Aβ clearance,^[^ ^40^ ^]^ we assessed the effect of UA on astrocytic Aβ engulfment using 3D reconstructions of GFAP and MX04. No differences were observed between 5×FAD+vehicle and 5×FAD+UA mice (Figure S6A,B, Supporting Information). We also examined the impact of UA on amyloidogenic APP processing.^[^ ^41^ ^]^ Immunoblot analysis revealed no significant difference in hippocampal expression of APP, APP‐CTFs, or β‐secretase BACE1 between vehicle‐ and UA‐treated 5×FAD mice (Figure S6C,D, Supporting Information). Collectively, these findings demonstrate that UA specifically enhances microglial phagocytosis of amyloid plaques in 5×FAD mice, without affecting astrocytic clearance or APP processing.

### 2.5 UA Induces Transcriptional Reprogramming in AD Microglia

**Figure 5:**
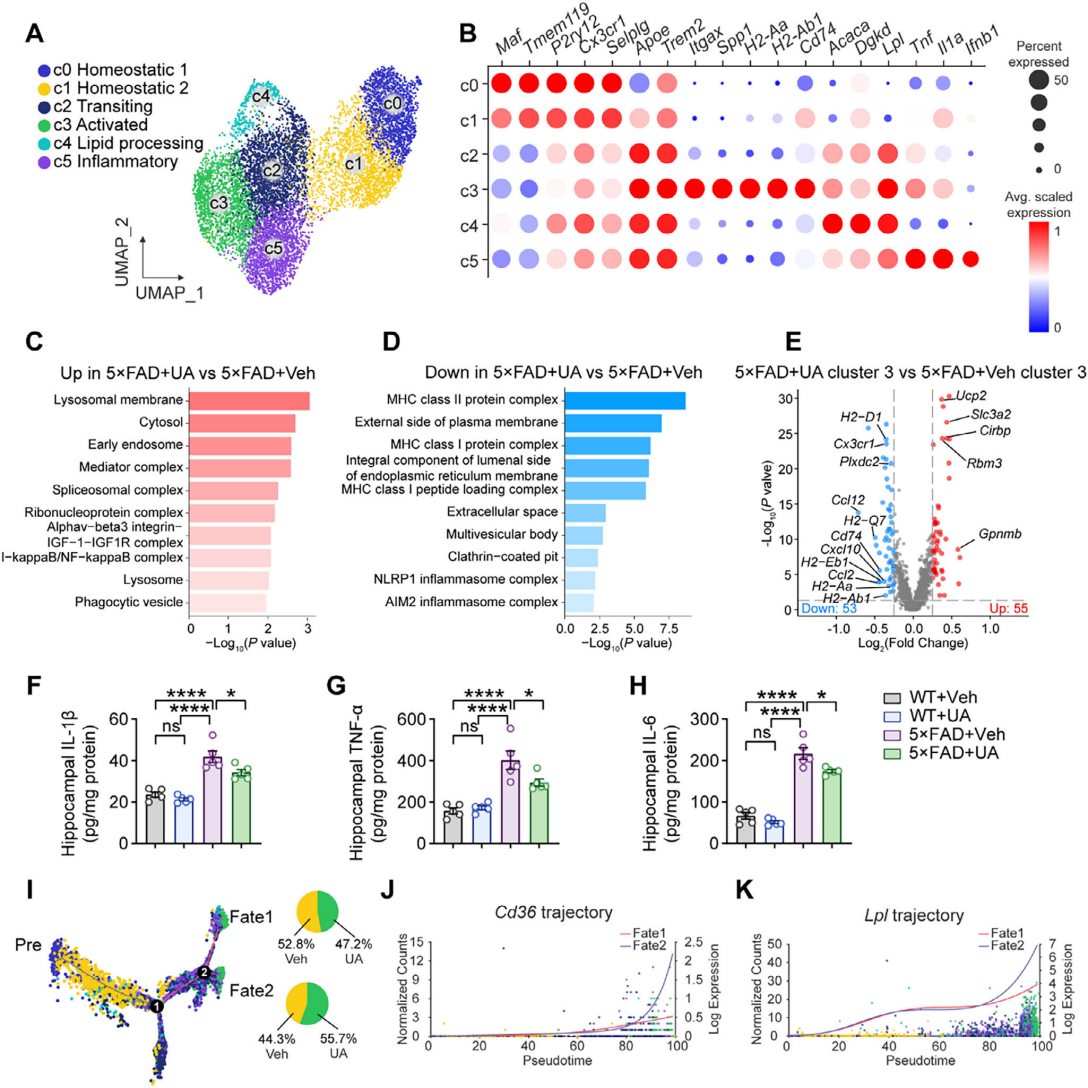
UA induces transcriptional reprogramming in AD microglia. A) Uniform manifold approximation and projection (UMAP) visualization of six distinct microglial clusters (c0‐c5) representing different transcriptional states in WT+Veh, 5×FAD+Veh, and 5×FAD+UA mice at 6.5 months of age. B) Bubble plots representation of signature gene expression patterns across identified microglial clusters. C,D) Gene Ontology (GO) enrichment analysis of total microglial differentially expressed genes (DEG) between 5×FAD+Veh and 5×FAD+UA mice, showing significantly upregulated (C) and downregulated (D) biological pathways. E) Volcano plots analysis of differentially expressed genes in cluster 3 comparing 5×FAD+UA versus 5×FAD+Veh groups. Differential expression analysis performed using Seurat function FindMarkers with Wilcoxon rank‐sum test (log_2_ fold change (FC) >0.25). *P* values were determined by Mann–Whitney U test with false discovery rate (FDR) correction. F,G,H) Quantification of hippocampal IL‐1β, TNF‐α and IL‐6 concentrations in WT+Veh, WT+UA, 5×FAD+Veh, and 5×FAD+UA groups (n = 5 per group). I) Pseudotime trajectory analysis of transcriptional dynamics based on key marker genes (detailed in Figure S7E, Supporting Information), with cells colored according to cluster identity from (A). J,K) Expression profiles of phagocytic genes *Cd36* and *Lpl* along the pseudotime trajectory. Data are presented as mean ± SEM. *P* values were determined by one‐way ANOVA followed by Tukey's *post hoc* analysis in (F–H). ns, not significant; ^*^ *P* < 0.05; ^****^ *P* < 0.0001.

To comprehensively analyze the influence of UA on microglial function in vivo, we performed single‐cell RNA sequencing (scRNA‐seq) analysis using hippocampal tissues from WT+vehicle, 5×FAD+vehicle, and 5×FAD+UA mice at ≈6.5 months using the 10X Genomics platform. Seurat analysis quality control yielded 16528 single cells, visualized using uniform manifold approximation and projection (UMAP; Figure S7A, Supporting Information). Unsupervised clustering based on cell‐type‐specific markers identified 9 distinct clusters across all samples (Figure S7B, Supporting Information). Further analysis of microglial populations revealed 6 distinct clusters comprising 9847 cells (**Figure** **5**A), characterized by distinct transcriptional signatures (Figure 5B).

Cluster 0 and 1, predominantly present in WT+vehicle mice (54.5% and 18.64% respectively; Figure S6C, Supporting Information), represented homeostatic microglia expressing high levels of *P2ry12* and *Cx3cr1* (Figure 5B). Cluster 1 displayed subtle activation compared to cluster 0, evidenced by decreased expression of homeostatic genes. Cluster 3, defined as activated response microglia (ARM), showed elevated expression of *Trem2*, *Apoe*, and MHC‐II presentation genes (*H2‐Aa, H2‐Ab1*, *and Cd74*). Cluster 2 exhibited an intermediate signature between homeostatic and ARM states, representing transitioning microglia. Cluster 4, a minor population, showed enrichment for lipid processing genes, while cluster 5 displayed high expression of inflammatory cytokines (*Il1a*, *Tnf*), indicating an inflammatory state. The distribution of these clusters remained similar between 5×FAD+vehicle and 5×FAD+UA mice (Figure S7C, Supporting Information). Quantitative Set analysis of Gene Expression (QuSAGE) revealed that cluster 3 exhibited marked upregulation of lysosomal and phagosomal pathways, concurrent with enhanced glycolysis and HIF‐1 pathway activation (Figure S7D, Supporting Information). Gene Ontology (GO) enrichment analysis demonstrated significant upregulation of pathways related to early endosome, lysosome, and phagocytic vesicle in 5×FAD+UA mice compared to 5×FAD+vehicle control mice (Figure 5C). Conversely, pathways involving MHC class I and II protein complexes and NLRP1 inflammasome complex were downregulated (Figure 5D).

Volcano plot analysis identified 55 upregulated and 53 downregulated differentially expressed genes (DEGs; log_2_ fold change > 0.25; *P* < 0.05) in 5×FAD+UA cluster 3 compared to 5×FAD+vehicle mice (Figure 5E). Notably, upregulated genes included anti‐inflammatory factors (*Gpnmb* and *Rbm3*) and neuroprotective genes (*Ucp2*, *Slc3a2*, and *Cirbp*). Downregulated genes included MHC class antigen presentation components (*H2‐Eb1*, *H2‐Ab1*, *Cd74*, and *H2‐D1*) and chemokines (*Ccl2*, *Cxcl10*, and *Ccl10*). Consistent with the scRNA‐seq findings, UA treatment significantly reduced hippocampal IL‐1β, TNF‐α, and IL‐6 levels in 5×FAD mice (Figure 5F–H), suggesting attenuated microglial immune responses following UA treatment. Pseudotime trajectory analysis based on differential gene expression (Figure S7E, Supporting Information) revealed two distinct cell lineage branches (Figure 5I). At branch point 2, UA treatment increased the proportion of microglia following fate 2, characterized by enhanced expression of phagocytic pathway genes (e.g., *Cd36, Lpl*; Figure 5J,K). These findings elucidated the molecular and functional adaptations in microglia following UA treatment, highlighting the role of UA in enhancing microglial phagocytic function while modulating inflammatory responses in AD mice.

### 2.6 UA Promotes Aβ Phagocytosis via Restoring Impaired Recycling of Phagocytic Receptors

**Figure 6:**
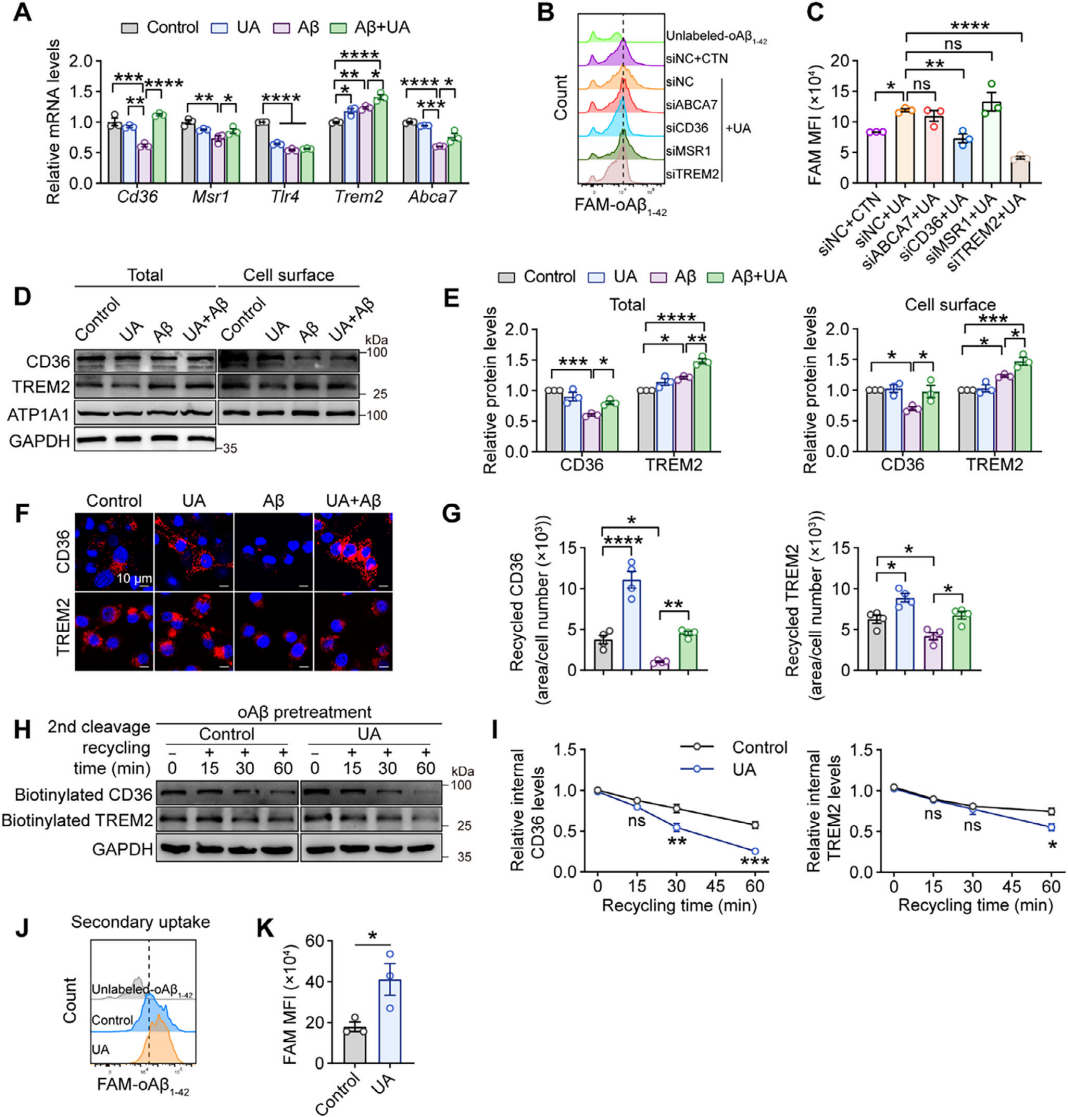
UA enhances recycling of intracellular phagocytic receptors in microglia. A) Quantitative RT‐PCR analysis of Aβ phagocytosis‐associated receptor transcripts in primary microglia following treatment with vehicle (control), UA, Aβ, or UA+Aβ (n = 3 independent experiments). B) Representative flow cytometry histograms showing FAM‐oAβ_1‐42_ (1 µm, 3 h) uptake in primary microglia following siRNA transfection (24 h) and/or UA treatment (100 µm, 12 h). C) Quantification of MFI from (B) (n = 3 independent experiments). D,E) Western blot analysis of total and cell surface CD36 and TREM2 expression in primary microglia following indicated treatments, assessed by surface biotinylation assay (n = 3 independent experiments). F) Representative confocal images showing receptor recycling for CD36 and TREM2 in primary microglia (scale bar, 10 µm). G) Quantification of receptors recycling from (F) (n = 4 independent experiments). H,I) Time‐course analysis of CD36 and TREM2 receptor recycling from intracellular compartments to the cell surface in primary microglia with/without UA treatment following oAβ_1‐42_ exposure (n = 3 independent experiments). J) Flow cytometry analysis of secondary FAM‐oAβ_1‐42_ uptake (1 µm, 3 h) following primary unlabeled‐oAβ_1‐42_ exposure (1 µm, 12 h). K) Quantification of MFI from (J) (n = 3 independent experiments). Data are the mean ± SEM. *P* values were determined by one‐way ANOVA followed by Tukey's *post hoc* analysis in (A, E, and G) and Dunnett's multiple comparisons test in (C), two‐way ANOVA followed by Sidak's multiple comparisons test in (I), and two‐tailed unpaired Student's *t*‐test in (K). ns, not significant; ^*^ *P* < 0.05; ^**^ *P* < 0.01; ***P < 0.001; ^****^ *P* < 0.0001.

Given the critical role of phagocytic receptors in microglial phagocytosis and clearance of Aβ,^[^ ^42^ ^]^ we examined the expression of key phagocytic receptors, including *Cd36*, *Msr1*, *Tlr4*, *Trem2*, *Abca7*, *Lrp1*, *Mertk*, *Itgb3*, and *Itgb5*.^[^ ^43^, ^44^, ^45^, ^46^ ^]^ Aβ exposure reduced receptor levels in microglia (**Figure** **6**A; Figure S8A, Supporting Information), suggesting compromised Aβ‐binding capacity. UA treatment counteracted Aβ‐induced reduction of *Cd36*, *Msr1*, and *Abca7*, while further enhancing *Trem2* expression (Figure 6A; Figure S8A, Supporting Information). The Aβ‐induced *Trem2* upregulation may be attributed to microglial activation.^[^ ^47^ ^]^ To identify the key receptors mediating UA‐enhanced phagocytosis, we performed in vitro phagocytosis assays in microglia following selective knockdown of *Cd36*, *Msr1*, *Trem2*, or *Abca7*. UA‐enhanced phagocytosis was specifically impaired by *Cd36* or *Trem2* knockdown, but not by *Msr1* or *Abca7* knockdown in primary microglia, suggesting essential roles for CD36 and TREM2 in UA‐enhanced microglial phagocytosis (Figure 6B,C). Given their established roles in LC3‐associated endocytosis and activation, we evaluated CD36 and TREM2 protein expression in primary microglia. Aβ treatment alone decreased the levels of total and surface CD36 while increasing TREM2 expression (Figure S8B–D, Supporting Information). Both biotinylation assay and flow cytometry confirmed that UA treatment increased total and surface expression of CD36 and TREM2 following Aβ exposure (Figure 6D,E; Figure S8E, Supporting Information).

We hypothesized that UA‐enhanced microglial phagocytosis may be attributed to altered receptor dynamics. Using an established receptor recycling assay (Figure S8F,G, Supporting Information) as previously described,^[^ ^48^ ^]^ we found that UA significantly alleviated the Aβ‐induced abrogation of CD36 and TREM2 recycling (Figure 6F,G). Consistent with this finding, UA decreased the levels of internalized biotinylated CD36 and TREM2 in primary microglia following Aβ treatment (Figure 6H,I), indicating enhanced recycling kinetics. UA‐induced receptor recycling was completely inhibited by probenecid, a broad inhibitor of organic anion transporters that blocks cellular UA entry, but not by NAC (Figure S8H–J, Supporting Information), indicating that UA enters microglia to exert intracellular regulatory effects independent of antioxidant mechanisms. To further evaluate whether UA improved receptor recycling capacity, we assessed microglial response to a secondary Aβ challenge. Following primary Aβ uptake for 24 h, microglia were exposed to FAM‐oAβ_1‐42_. UA‐treated cells showed an ≈130% increase in FAM fluorescence intensity (Figure 6J,K). Compared to the modest increase (≈15%) in initial Aβ uptake (Figure 4I), this substantial enhancement in secondary uptake suggests that receptor recycling plays a critical role in UA‐enhanced phagocytosis. Collectively, these findings demonstrate that UA enhances microglial phagocytic capacity primarily by restoring CD36 and TREM2 recycling that is compromised by Aβ exposure.

### 2.7 UA Enhances Microglial Aβ Degradation via Lysosomal Pathway

**Figure 7:**
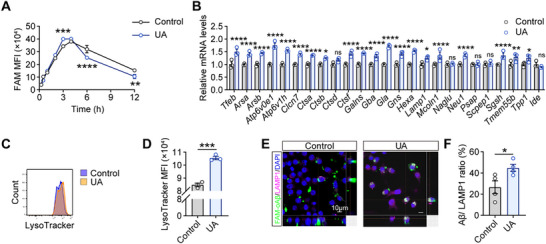
UA enhances microglial Aβ degradation through the lysosomal pathway. A) Temporal analysis of intracellular Aβ clearance in primary microglia pretreated with vehicle (control) or UA (100 µm, 12 h) followed FAM‐oAβ_1‐42_ (1 µm) exposure. MFI quantified by flow cytometry at indicated time points (15 min to 12 h; n = 3 independent experiments). B) Quantitative RT‐PCR analysis of lysosomal biogenesis regulator and Aβ‐degrading enzymes transcripts in primary microglia following UA treatment (100 µm, 6 h) versus vehicle control (n = 3 independent experiments). C) Representative flow cytometry histograms showing LysoTracker Red fluorescence intensity in UA‐treated versus vehicle control microglia. D) Quantification of MFI from (C) (n = 3 independent experiments). E,F) Representative confocal images (E) and quantification (F) of the co‐localization between FAM‐oAβ_1‐42_ (green) and LAMP1‐positive lysosomes (red) (n = 4 independent experiments). Scale bar, 10 µm. Data are presented as mean ± SEM. *P* values were determined by two‐way ANOVA followed by Sidak's multiple comparisons test in (A) and two‐tailed unpaired Student's *t*‐test in (B, D, and F). ns, not significant; ^*^ *P* < 0.05; ^**^ *P* < 0.01; ^***^ *P* < 0.001; ^****^ *P* < 0.0001.

Following our observations of enhanced Aβ uptake, we explored the influence of UA on subsequent Aβ degradation in microglia. Using a flow cytometry‐based pulse‐chase assay,^[^ ^43^ ^]^ we monitored intracellular FAM‐oAβ_1‐42_ fluorescence intensity. UA‐treated primary microglia showed two distinct features in Aβ processing: they reached peak mean fluorescence intensity (MFI) more rapidly than controls, and exhibited accelerated fluorescence decline, suggesting enhanced Aβ uptake and clearance capabilities (**Figure** **7**A). Similar degradation kinetics were observed in BV2 microglial cells (Figure S9A, Supporting Information). Given that intracellular Aβ degradation occurs through multiple pathways, including the ubiquitin–proteasome, autophagy–lysosome, proteases, and endosome–lysosome systems,^[^ ^49^ ^]^ we examined the expression of key degradation enzymes involved in these pathways, including *Ctsb*, *Ctsd*, *Tfeb*, *Lamp1*, *Lamp2*, and *Ide*. UA treatment upregulated lysosomal genes (*Ctsb*, *Ctsd, Tfeb*, and *Lamp1*) in both primary microglia and BV2 cells (Figure 7B; Figure S9B, Supporting Information). This upregulation was maintained even in the presence of Aβ (Figure S9C, Supporting Information), suggesting enhanced lysosomal function. Furthermore, we performed LysoTracker Red staining, which revealed enhanced lysosomal biogenesis following UA administration (Figure 7C,D). Additionally, immunofluorescence analysis of FAM‐oAβ_1‐42_‐treated cells demonstrated increased colocalization between Aβ and LAMP1 in UA‐treated microglia (Figure 7E,F). Collectively, these findings demonstrate that UA enhances microglial Aβ clearance by promoting lysosomal degradation pathway activity.

## 3 Conclusion

**Figure 8:**
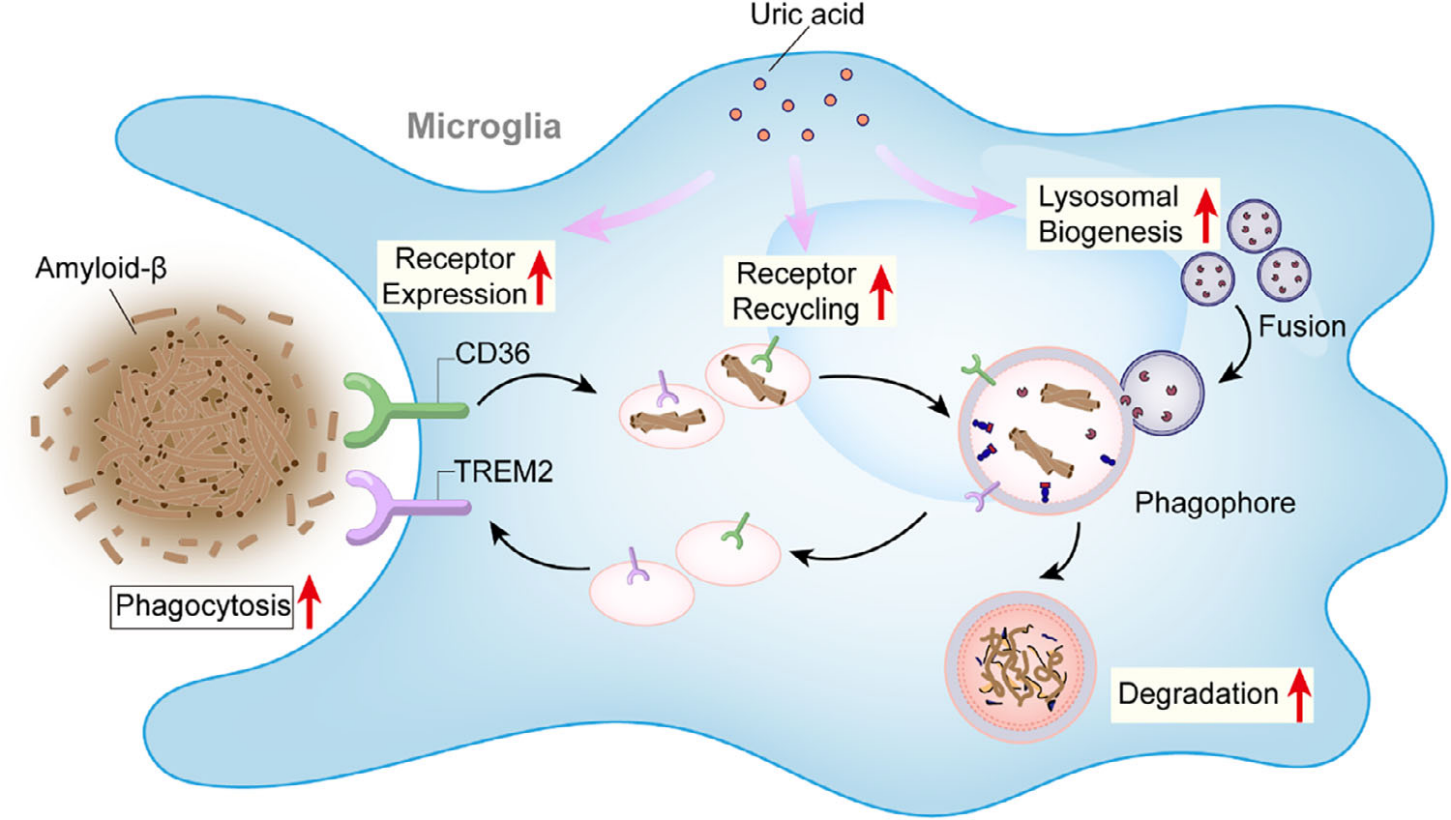
UA is an endogenous modulator of microglial function and a novel target for therapeutic intervention in AD. Epidemiological studies associate higher serum UA levels with reduced AD risk. Mechanistically, UA enhances microglial phagocytic capacity primarily by restoring CD36 and TREM2 receptor recycling that is compromised by Aβ exposure. Therapeutically, UA supplementation improves cognitive function and reduces neuropathology in the AD mouse model by enhancing microglial Aβ clearance.

AD remains a significant public health burden in our aging society due to the lack of effective interventions to halt its progression. Our study provides novel insights into the microglial regulation and neuroprotective potential of UA in AD (**Figure** **8**). We observed that lower SUA levels are associated with increased risk of AD and accelerated cognitive decline. These findings are consistent with previous studies reporting diminished SUA levels in AD patients and a protective effect of higher baseline SUA against incident dementia.^[^ ^23^, ^25^, ^50^, ^51^ ^]^ Conversely, some studies have linked elevated SUA levels to heightened risk of vascular or mixed dementia and altered acetylcholinesterase activity,^[^ ^52^, ^53^ ^]^ indicating that the relationship between UA levels and AD is complex and may involve multiple factors.

Given the evolutionary loss of uricase, physiological SUA levels are significantly higher in humans compared to mice. To avoid hyperuricemia‐related complications, we administered UA at a dose of 200 mg kg^−1^ daily for two months, as previous described.^[^ ^39^ ^]^ Our dosing regimen maintained mildly elevated ISF UA levels for ≈9 h without significant effects on neuroinflammation or renal function, differing from previous methods involving stereotactic injection or uricase (*Uox)*‐knockout strategies.^[^ ^54^, ^55^ ^]^ These results suggest that our protocol is physiologically relevant and reliable for studying the effects of UA in mice despite interspecies differences in SUA levels. Future studies should incorporate rigorous monitoring for potential hyperuricemia‐related effects during UA supplementation for periods longer than two months. Considering the inherent differences in blood‐brain barrier (BBB) properties and UA transport mechanisms between humans and mice, which may influence cerebral UA metabolism, a more comprehensive investigation into the relationship between AD and CSF or serum UA is warranted. Subsequent studies should evaluate optimized urate‐elevation therapies, such as oral inosine supplementation or targeted intracerebral delivery approaches. Implementation of rigorous monitoring protocols and well‐defined experimental cohorts is crucial, particularly since the beneficial effects of UA have been observed in patients without hyperuricemia and with moderately elevated baseline SUA levels.^[^ ^56^ ^]^

Epidemiological studies consistently demonstrate reduced SUA levels in AD patients, yet the biological basis remains poorly understood. Based on current evidence, we propose several potential mechanisms that warrant further validation: 1) impaired purine metabolism through reduced xanthine oxidase activity or upstream disturbances,^[^ ^57^ ^]^ 2) accelerated oxidative degradation, given UA's role as a major endogenous antioxidant in the high‐oxidative‐stress AD environment, 3) enhanced renal/cerebral excretion via overexpressed ABCG2 and MRP4 transporters reported in AD brain,^[^ ^58^, ^59^ ^]^ and 4) *APOE4* genotype association with lower SUA levels compared to *APOE2* carriers.^[^ ^60^ ^]^

Our findings demonstrate that UA enhances microglial clustering around amyloid plaques, promoting Aβ uptake and subsequent degradation. This effect is particularly relevant in the early stages of AD when microglia are involved in Aβ clearance but exhibit declining phagocytic and scavenging efficiency with age.^[^ ^31^, ^61^ ^]^ Insufficient microglial capacity contributes to excessive Aβ accumulation in the brain.^[^ ^62^ ^]^ As AD progresses, microglia exhibit massive proliferation and heightened inflammatory activation but reduced phagocytic ability.^[^ ^63^ ^]^ Notably, UA downregulated proinflammatory genes associated with MHC class II antigen presentation (*H2‐Eb1*, *H2‐Ab1*, *Cd74*, and *H2‐D1*) and chemokines (*Ccl2*, *Cxcl10*, and *Ccl10*) in activated response microglia (ARM), a subtype enriched in AD risk genes and localized around amyloid plaques.^[^ ^64^ ^]^ This suggests that UA modulates microglial immune responses, potentially restoring their phagocytic capacity. A recent study has demonstrated that UA serves as a TFEB activator, thereby promoting microglial autophagy and enhancing Aβ clearance.^[^ ^65^ ^]^ Furthermore, UA has been reported to suppress microglial inflammatory cytokine production and protect dopaminergic neurons in Parkinson's disease.^[^ ^39^ ^]^ Given that UA accounts for 60% of total plasma antioxidant capacity, it may protect the brain from Aβ toxicity and glial neuroinflammation; however, this hypothesis warrants further investigation.

A limitation of our study is the lack of human *APOE* isoforms in the 5×FAD mice, especially considering that 56.5% of AD participants in our study were *APOE4* carriers (Table S1, Supporting Information). Previous research indicates that APOE4‐expressing microglia display enhanced proinflammatory gene expression and impaired Aβ phagocytosis compared to *APOE3* microglia, with reduced colocalization with amyloid plaque deposits.^[^ ^66^, ^67^ ^]^ Additionally, higher SUA levels have been shown to benefit cognitive function in mild cognitive impairment (MCI) patients carrying the *APOE4* allele.^[^ ^68^ ^]^ These findings suggest that the effects of UA on microglial function may be influenced by *APOE* genotype. Therefore, future studies should utilize models expressing human *APOE* isoforms to investigate how different *APOE* subtypes modulate UA's impact on microglial Aβ clearance.

Mechanistically, we observed that UA treatment mitigated oligomeric Aβ‐induced downregulation of phagocytic receptors, including CD36, MSR1, and ABCA7, aligning with previous observations in AD mice.^[^ ^31^ ^]^ In vitro phagocytosis assays revealed that UA enhances Aβ phagocytosis by restoring impaired recycling of CD36 and TREM2. Phagocytic receptor's recycling depends on the retromer complex, Rab GTPases, retromer, ESCRT components, and specific phosphoinositide signaling. Reduced expression of VPS35, a key component of the retromer, has been demonstrated in microglia from AD patients, exacerbating neuropathology in *Cx3cr1*‐Cre; *VPS35*;5×FAD mice.^[^ ^69^, ^70^ ^]^ Therefore, UA may enhance the expression of recycling mediators, improving the dynamics and phagocytic ability of these receptors. The precise mechanisms by which UA affects CD36 and TREM2 recycling require further elucidation.

In conclusion, our study identifies UA as an endogenous modulator of microglial function and Aβ processing in AD. We demonstrate that UA supplementation improves cognitive function and reduces amyloid plaque deposition in an AD mouse model. Mechanistically, UA modulates microglial immune responses and restores the recycling of key phagocytic receptors, suggesting its potential as a therapeutic strategy for AD.

## 4 Experimental Section

### Subject and Clinical Assessment

Data used for this study were obtained from the ADNI‐1 cohort, accessed through the ADNI database (adni.loni.usc.edu).^[^ ^25^ ^]^ Disease severity was assessed using the CDR scale, a validated instrument widely implemented in longitudinal studies and clinical trials of AD. The CDR provides a composite dementia severity score derived from a weighted algorithm that emphasizes memory domain performance while incorporating other cognitive domains. Scores range from 0 (cognitively normal) to 3 (severe dementia), with intermediate ratings of 0.5 (questionable dementia), 1 (mild dementia), and 2 (moderate dementia).^[^ ^71^ ^]^ From the total ADNI‐1 cohort, serum UA measurements were available for 200 participants, comprising 76 subjects with CDR = 0 and 124 subjects with CDR ≥ 0.5. Baseline SUA data were extracted from the Biospecimen section of the ADNI database (accessed March 21, 2022). Detailed metabolomic analysis protocols are available through the ADNI database (https://ida.loni.usc.edu/pages/access/studyData.jsp).

### Animals

This study used both male and female C57BL/6J wild‐type (WT) mice and 5×FAD (APP^Sw, Fl, Lon^, PS‐1^M146L, L286V^) mice (Jackson Laboratory, stock number 34840‐JAX).^[^ ^72^ ^]^ All animals were maintained at the Xiamen University Laboratory Animal Center (China) under standard laboratory conditions (12 h‐light/dark cycle, controlled temperature and humidity) with ad libitum access to water and standard rodent chow.

5×FAD mice and their WT littermates were treated with either vehicle or UA (200 mg kg^−1^, *i.p*.) daily from 4 to 6 months of age. The UA dosage was selected based on previous pharmacological studies,^[^ ^39^ ^]^ with careful consideration of species differences in UA metabolism, particularly since humans lack functional uricase.^[^ ^35^ ^]^ This treatment regimen was designed to achieve physiologically relevant serum UA levels that model the UA deficiency observed in AD patients, thereby enabling evaluation of UA supplementation as a potential therapeutic strategy.

At 6 months of age, all mice underwent behavioral assessments. Following behavioral testing, animals were anesthetized with 2% isoflurane for orbital sinus blood collection. Mice were then euthanized by cervical dislocation, and brains were rapidly harvested and bisected sagittally. One hemisphere was flash‐frozen and stored at −80 °C for protein analysis, while the contralateral hemisphere was fixed in 4% paraformaldehyde for immunofluorescence studies.

Behavioral analyses included 20–24 mice per experimental group (10–12 males, 10–12 females), while subsequent in vivo analyses utilized only male mice. All experimental procedures were conducted in accordance with protocols approved by the Institutional Animal Care and Use Committee (Animal Ethics Approval: XMULAC20240246) and complied with Xiamen University Laboratory Animal Center guidelines.

### Chemicals and Reagents

Uric acid (Sigma, U2625) and probenecid (MCE, HY‐B0545) were obtained from the respective suppliers. Aβ_1‐42_ peptide and FAM‐labeled Aβ_1‐42_ peptide were purchased from GL Biochem (Shanghai, China). Primary and secondary antibodies used in Western blot, immunofluorescence, and flow cytometry analyses are detailed in Table S3 (Supporting Information). For in vivo studies, UA was suspended in a 0.5% sodium carboxymethylcellulose vehicle for intraperitoneal injection. For in vitro experiments, UA stock solutions (15 mg mL^−1^) were prepared in 0.5 m NaOH, filter‐sterilized, and diluted in culture medium.

### Biochemical Measurements

Serum levels of uric acid (UA; C012‐2‐1), alanine aminotransferase (ALT; C009‐2‐1), aspartate aminotransferase (AST; C010‐2‐1), alkaline phosphatase (ALP; A059‐1‐1), albumin (A028‐2‐1), total protein (A045‐2‐2), blood urea nitrogen (BUN; C013‐1‐1), creatinine (C011‐2‐1), total cholesterol (A111‐1‐1), triglycerides (A110‐1‐1), creatine kinase (CK; A032‐1‐1), and fasting blood glucose (A154‐1‐1) were determined using commercial biochemical assay kits (Nanjing Jiancheng Bioengineering Institute, Nanjing, China) according to the manufacturer's instructions.

### In Vivo Microdialysis

In vivo microdialysis, an established technique for sampling ISF from conscious, freely moving animals,^[^ ^73^ ^]^ was employed to evaluate hippocampal ISF UA dynamics following peripheral administration in both 4‐month‐old WT and 5×FAD mice.

Surgical procedure. Four‐month‐old mice were anesthetized with 2% isoflurane and stereotaxically immobilized (RWD, Shenzhen, China). After creating a burr hole at predetermined coordinates (anteroposterior: ‐3.1 mm, mediolateral: ‐2.5 mm), a stainless‐steel guide cannula (MAB 10.8.2, Sweden) was stereotaxically implanted into the left hippocampus (dorsoventral: ‐1.2 mm, 12° angle) and secured with dental acrylic cement. After cannulation, animals were individually housed and allowed a minimum of one‐week recovery period.

Microdialysis. Following recovery, mice were briefly anesthetized and fitted with a protective collar. The guide cannula stylet was removed, and a pre‐equilibrated microdialysis probe (MAB 10.8.2.PES, molecular weight cutoff: 6 kDa) was carefully inserted through the guide cannula. Animals were then transferred to observation chambers (CMA402, Sweden), allowing unrestricted movement. The probe was connected to a syringe pump delivering artificial CSF comprising: 2.7 mm KCl, 140 mm NaCl, 1.2 mm CaCl_2_, 1.0 mm MgCl_2_, 0.3 mm NaH_2_PO_4_, and 1.7 mm Na_2_HPO_4_; pH 7.2. Perfusion was maintained at a constant flow rate of 1 µL min^−1^. Following UA administration, dialysate samples were collected at 60‐min intervals over 12 h using a refrigerated fraction collector (MAB 85). Samples were stored at −80 °C for subsequent analysis.

### UA Analysis

Quantitative analysis of UA in microdialysate samples was performed using an Ultimate 3000 ultra‐high‐performance liquid chromatography (UHPLC) system coupled to a Q‐Exactive Hybrid Quadrupole‐Orbitrap mass spectrometer (Thermo Fisher Scientific, Waltham, MA, USA) equipped with an electrospray ionization (ESI) source operating in negative‐ion mode. Chromatographic separation was achieved using a Luna C8 analytical column (150 mm × 2.0 mm, 5 µm particle size; Phenomenex, Torrance, CA, USA). The mobile phases consisted of 5 mm ammonium acetate/0.1% acetic acid (pH 4.2) (A) and methanol (B). The flow rate was maintained at 0.4 mL min^−1^ with a 1:3 split ratio before entering the mass spectrometer. The gradient elution program was as follows: 5–25% B from 0 to 10 min, 25–100% B from 10 to 15 min, and 100%–5% B from 15 to 16 min (column re‐equilibration); the mobile phase was then held at 5% B until 20 min. Mass spectrometric analysis was performed in full‐scan mode across a mass range of m/z 100–500. Target ion identification was based on *m/z* values obtained from UA reference standards. Sample injection volume was 5 µL. Instrument control and data processing were performed using Xcalibur 4.1 software (Thermo Fisher Scientific).

### Behavioral Tests

Cognitive and memory functions were evaluated in 5×FAD and WT littermates following 2‐month treatment with either vehicle or UA (n = 20–24 mice per group, with equal numbers of males and females). Prior to testing, animals were habituated to the experimental room for a minimum of 30 min. All mice were maintained in their home cages and handled by the tail base to minimize stress. Behavioral tests were sequentially administered in order of increasing stress: novel object recognition (NOR) test, Y‐maze test, and Morris water maze (MWM) test. To ensure experimental rigor, all behavioral assessments were performed by investigators blinded to the experimental groups and treatment conditions.

NOR test. The NOR paradigm comprised three sequential phases over three days. On day 1 (habituation phase), each animal freely explored the empty arena for 10 min. During the familiarization phase (day 2), mice were exposed to two identical objects placed in opposite corners of the arena for 10 min. In the test phase (day 3), one familiar object was replaced with a novel object, and mice were allowed 10‐min of exploration while their movements were recorded. Object exploration was defined as nose‐directed behavior within 2 cm of the object. Behavioral recordings were analyzed using EthoVision XT 14.0 (Noldus, Wageningen, The Netherlands) to calculate discrimination indices. The apparatus and objects were cleaned with 70% ethanol between trials.

Y‐maze test. Spontaneous alternation behavior was assessed in a symmetrical Y‐maze with three identical arms (40 cm × 10 cm × 16 cm) positioned at 120° angles and labeled A, B, and C. Following established protocols,^[^ ^74^ ^]^ mice were placed into arm A facing the center and allowed 10‐min free exploration. Video recordings were analyzed to determine the number of alternations, with the alternation percentage calculated as: (number of alternations)/(total arm entries – 2) × 100. The apparatus was sanitized with 70% ethanol between subjects to eliminate olfactory cues.

MWM test. Spatial learning and memory were evaluated using the MWM paradigm in a circular pool (diameter: 120 cm; depth: 50 cm) filled with opaque water maintained at 22 °C,^[^ ^75^ ^]^ with a hidden platform submerged 1 cm below the water surface in the center of one quadrant. The acquisition phase consisted of six consecutive days of training, with mice performing two daily trials from different starting positions. Each trial had a 60‐s maximum duration; if unsuccessful, mice were guided to the platform for a 10‐s orientation period. Mice were towel‐dried between trials. On day 7, a probe trial was conducted by removing the platform and allowing mice a 60‐s free swim starting from the quadrant opposite to the previous platform location. The EthoVision XT 14.0 system (Noldus, Wageningen, The Netherlands) recorded and analyzed multiple parameters, including escape latency, time spent in each quadrant, platform crossings, and swim velocity throughout all testing phases.

### Immunofluorescence Analysis

Brain tissue processing and imaging. Following 2‐month vehicle or UA treatment, 5×FAD mice received intraperitoneal methoxy‐X04 (MX04, Tocris, 4920; 10 mg kg^−1^) 3 h prior to transcardial perfusion with ice‐cold PBS. Harvested brain tissues underwent gradient sucrose dehydration (3 days) before optimal cutting temperature (OCT) embedding. Coronal sections (20 µm) were prepared and either immediately processed or stored at −80 °C. For immunohistochemistry, sections were fixed and blocked (10% goat serum, 0.2% Triton‐X in PBS; 1 h, room temperature) before primary antibody incubation. Sections were immunolabeled overnight (4 °C) with anti‐Aβ (6E10, Biolegend) and anti‐Iba1 (Wako). After PBS washes, sections were incubated with Alexa‐fluorophore‐conjugated secondary antibodies (Invitrogen; 2 h, room temperature), counterstained with DAPI (Sigma, 1 µg mL^−1^; 10 min), and mounted using anti‐fade mounting medium (LABLEAD Inc.).

Images were acquired using an Olympus FV1000MPE‐B, Leica Aperio Versa 200, or Evident FV3000 confocal microscope. For plaque‐associated microglia analysis, z‐stacks (12 µm depth; 16 optical slices; 0.75 µm intervals) were captured. 3D reconstruction utilized Imaris software (v.10.0.0; Bitplane) for surface rendering of microglia and MX04‐positive dense‐core plaques, distance measurement between Iba1 surface centroids and the nearest plaque edge (MATLAB), and quantification of plaque‐associated microglia (≤15 µm from plaque edge). Intracellular Aβ quantification employed the generation of Iba1/GFAP‐masked channels, surface reconstruction of internalized amyloid plaques, and normalization of internalized Aβ signals to total glial cell volume. Image analysis and quantification were conducted by investigators blinded to the experimental conditions.

Cell culture imaging. Receptor recycling analysis followed procedures detailed in the “Receptor recycling assay” section. For Aβ‐lysosome colocalization studies, cells were treated with FAM‐oAβ_1‐42_ (1 µm, 3 h) ± UA (6 h), fixed with 4% PFA, and immunolabeled with anti‐LAMP1 (1:400; Abcam, ab25630), anti‐CD36 (1:100; Novusbio, NB400‐144), or anti‐TREM2 (1:100; R&D systems, 17 291) at 4 °C overnight. Sections were then incubated with Alexa Fluor 594 secondary antibodies (1:1000; Invitrogen) at room temperature for 1 h. Images were acquired using a Nikon A1R Plus confocal microscopy and quantified using ImageJ (NIH). Image analysis and quantification were conducted by investigators blinded to experimental conditions.

### ELISA

Cerebral cortex tissues were homogenized in RIPA lysis buffer (25 mm Tris‐HCl, pH 7.6, 150 mm NaCl, 1% NP‐40, 1% sodium deoxycholate, 0.1% SDS, and protease inhibitor cocktail). Soluble Aβ_42_ levels were quantified using a human Aβ_42_ ELISA kit (Thermo Fisher Scientific, KHB3441) according to the manufacturer's instructions. For inflammatory cytokine detection, hippocampal tissue homogenates and mouse serum were analyzed using commercial ELISA kits according to the manufacturer's instructions (IL‐1β: E‐EL‐M0037; IL‐6: E‐EL‐M0044; and TNF‐α: E‐EL‐M3063; all from Elabscience).

### Western Blot Analysis

Briefly, brain tissue samples were homogenized in RIPA lysis buffer, incubated at 4 °C for 20 min, and centrifuged (12000 ×g, 15 min). Protein concentration was determined using a bicinchoninic acid protein assay kit (Beyotime). Soluble protein extracts (40 µg) were resolved using 16.5% Tris‐Tricine‐SDS polyacrylamide gels (Solarbio) and electrophoretically transferred to PVDF membranes. After blocking, membranes were immunoblotted overnight at 4 °C with primary antibodies, then incubated with HRP‐conjugated secondary antibodies (goat anti‐rabbit or goat anti‐mouse IgG H&L; Bioss) for 2 h at room temperature. Immunoreactive bands were visualized using an Azure C300 chemiluminescent imaging system (USA) and quantified by densitometric analysis using ImageJ software (NIH).

### Microglial Cell Culture

BV2 microglial cells (RRID: CVCL_0182) were purchased from the Kunming Cell Bank, Chinese Academy of Sciences (Kunming, China). Cell authenticity was verified by the supplier using DNA profiling of polymorphic short tandem repeat (STR) markers, confirming the absence of cross‐contamination with other cell lines. Additionally, all cell cultures tested negative for mycoplasma and bacterial contamination throughout the study duration. Cells were maintained in DMEM supplemented with 10% FBS and antibiotics (100 U mL^−1^ penicillin G, 100 µg mL^−1^ streptomycin) at 37 °C in a 5% CO_2_ humidified incubator. Primary microglial cultures were established following previously described protocols.^[^ ^76^ ^]^ Briefly, neonatal mouse brains, following meningeal removal, were enzymatically dissociated using papain (8 U mL^−1^), and the resulting cell suspension was filtered through a 70‐µm cell strainer before seeding in poly‐D‐lysine‐coated T75 flasks. After a 3‐day initial culture period, the medium was supplemented with GM‐CSF (30 ng mL^−1^; Biolegend) and refreshed every 3 days thereafter. After astrocytes reached complete confluency, loosely adherent microglia were harvested by gentle agitation and subsequently seeded into 6‐well plates or glass‐bottom confocal dishes for experimental procedures.

### Oligomeric Aβ Preparation

Aβ_1‐42_ oligomers were prepared using both FAM‐labeled and unlabeled peptides following a standardized protocol. Initially, 1 mg of Aβ_1‐42_ powder was dissolved in 1 mL hexafluoroisopropanol (HFIP) and allowed to evaporate overnight at room temperature to form a peptide film. The resultant film was reconstituted in DMSO to a concentration of 5 mm and sonicated in a water bath for 10 min. The DMSO‐peptide solution was subsequently diluted in DMEM/F‐12 medium to final concentrations of 100 µm for cell culture applications and 1 mm for oligomerization analysis. Oligomeric Aβ_1‐42_ preparations were generated through overnight incubation at 4 °C, and the oligomers were stored at −80 °C until use.

### In Vivo Aβ Phagocytosis Assay

Following 2‐month UA treatment, 6‐month‐old WT and 5×FAD mice (5×FAD+Veh, 5×FAD+UA, and WT controls) received intraperitoneal injections of MX04 (Tocris, 4920; 10 mg kg^−1^ in 9:1 PBS: DMSO) for 3 h. Following PBS perfusion, isolated hippocampi were mechanically dissociated and enzymatically digested (37 °C, 30 min) in a solution containing papain (1 mg mL^−1^; Solarbio, G8430), DNase I (20 U mL^−1^; Solarbio, D8071), and collagenase IV (100 U mL^−1^; ThermoFisher, 17 104 019). The digested tissue was gently triturated and filtered through a 70‐µm cell strainer to obtain a single‐cell suspension, which was then centrifuged (500 ×g, 4 °C, 5 min, reduced brake) after D‐Hanks washing. Cell pellets were resuspended in 30% stock isotonic Percoll (SIP) solution and layered over 70% SIP solution for density gradient centrifugation (650 ×g, room temperature, 30 min, acceleration setting 3, brake setting 0). Mononuclear cells isolated from the 30/70 interface were incubated with CD16/CD32 Fc receptor blocking antibody (BD Bioscience, 553 141) and then labeled with APC‐eFluor 780‐CD45 (Invitrogen, 47‐0451‐82), Percp‐Cy5.5‐CD11b (BD, 550 993), and fixable viability dye (FVD, Invitrogen, 65–0867; 1:1000) in FACS buffer (2% FBS/PBS with 1 mM EDTA) for 1 h at 4 °C. After three washes, cells were analyzed by flow cytometry, with debris excluded using forward/side‐scatter parameters and live cells selected as FVD‐negative. MX04⁺CD11b⁺CD45^int^ microglia were quantified as shown in Figure S5A (Supporting Information), with MX04‐injected WT mice serving as negative controls.

### In Vitro Phagocytosis Assay

Primary mouse microglial cells were seeded in 6‐well plates and pre‐treated with UA (100 µm) or vehicle (control) for 12 h prior to exposure to oligomeric FAM‐labeled Aβ_1‐42_ (1 µm) for 3 h. Following oAβ_1‐42_ treatment, culture medium was removed, cells were washed with PBS, and fixed with 4% paraformaldehyde for subsequent analysis by flow cytometry or fluorescence microscopy (Olympus IX51) to determine mean fluorescence intensity (MFI). For unlabeled‐oAβ_1‐42_ uptake studies, fixed cells were permeabilized, blocked, and immunolabeled with anti‐Aβ antibody (6E10) overnight, followed by secondary antibody incubation for immunofluorescence microscopy and FACS analysis. Sequential uptake assessment involved initial treatment with FAM‐labeled oAβ_1‐42_ (1 µm, 3 h) followed by oAβ_1‐42_ exposure for 24 h before analysis as described above. General endocytic capacity was evaluated using FITC‐labeled dextran (1 µg mL^−1^, 3 h) with flow cytometric detection.

### Flow Cytometry Analysis

Flow cytometric analyses of primary mouse microglia and BV2 cells were conducted using a Beckman CytoFlex instrument. All uptake and degradation assays utilized fixed cells to preserve intracellular fluorescence signals, while CD36 surface expression was evaluated using fixed, non‐permeabilized cells. For lysosomal assessment, live cells were stained with LysoTracker Red probes (75 nm, 30 min). Each sample analysis included a minimum of 10 000 events, with data processing performed using FlowJo software (v10.8.1, Tree Star).

### Single‐Cell RNA Sequencing Analysis

Sample preparation. Hippocampal tissues were collected from three experimental groups (WT, 5×FAD+vehicle, and 5×FAD+UA mice), with each sample comprising pooled hippocampi from three individual mice. The tissues underwent sequential enzymatic and mechanical dissociation to generate single‐cell suspensions. Myelin debris was eliminated using myelin removal beads (Miltenyi Biotec), and the resulting single cells were suspended in a 0.5% BSA solution for subsequent scRNA‐seq analysis.

Data processing. scRNA‐seq data were preprocessed using the NovelBrain Cloud Analysis Platform (www.novelbrain.com). Initial quality control filtering retained cells expressing more than 200 genes and with mitochondrial UMI rates below 20%, followed by removal of mitochondrial genes from the expression matrix. Data normalization and scaling were performed using the *Seurat* package (v4.1.1), incorporating UMI counts and mitochondrial percentage for regression.

Principal component analysis (PCA) was conducted using the top 2000 highly variable genes, with the first 10 principal components utilized for UMAP dimensionality reduction and graph‐based unsupervised clustering. Marker gene identification was performed using the FindAllMarkers function with the Wilcox rank‐sum test (criteria: Log_2_FC > 0.25, *P* < 0.05, minimum percentage > 0.1). Following doublet removal, the data underwent re‐scaling and re‐clustering for final analysis.

Differentially expressed genes (DEGs) were functionally characterized using Gene Ontology (GO) analysis (v2.5.13) in R, utilizing annotations from the GO database. Relative activation of gene sets across clusters was assessed using QuSAGE (v2.16.1), with statistical significance determined by Fisher's exact test. Temporal gene expression dynamics were analyzed using Monocle2 to construct pseudo‐time differentiation trajectories of hippocampal microglia, incorporating variable genes identified by Shaul et al. as ordering genes to map AD progression.^[^ ^77^ ^]^ Branch Expression Analysis Modeling (BEAM) was employed to identify genes determining cell fate decisions along trajectory branch points.

### RNA Isolation and Quantitative Real‐time PCR Analysis

Mouse primary microglia and BV2 cells were cultured in 6‐well plates and assigned to experimental groups. Total RNA was isolated using RNA Extraction Kits (Sangon, China) according to the manufacturer's instructions, and 1000 ng of total RNA was reverse transcribed using an Evo M‐MLV RT Mix Kit (Accurate Biology, AG11728). Quantitative PCR was performed using SYBR Green Mix (SparkJade Biotechnology, AH0104‐B) with the following thermal cycling profile: initial denaturation and polymerase activation at 95 °C for 5 min, followed by 40 cycles of denaturation (95 °C, 10 s), annealing (72 °C, 20 s), and extension (60 °C, 20 s), as previously described.^[^ ^78^ ^]^ Primer sequences are provided in Table S4 (Supporting Information).

### siRNA

The siRNAs were synthesized by Tsingke Biotech Co. (Beijing, China). The target sequences were as follows: CD36: 5′‐GGATCTGAAATCGACCTTA‐3′; MSR1: 5′‐CGACCTTATAGACACGGAA‐3′; ABCA7: 5′‐GGATTAGTGCTTAAGCTA‐3′; TREM2: 5′‐CGTTCTCCTGAGCAAGTTT‐3′. Transfection of primary microglial cells was performed using Lipofectamine 2000 reagent (Invitrogen) according to the manufacturer's instructions.

### Cell Surface Biotinylation Assay

Cell surface protein biotinylation was performed as previously described.^[^ ^79^ ^]^ Cells were washed with ice‐cold CM‐PBS (PBS supplemented with 1 mm CaCl_2_ and 1 mm MgCl_2_), followed by two sequential 20‐min incubations with ice‐cold Sulfo‐NHS‐LC‐biotin (0.5 mg mL^−1^, Apexbio, A8003). The biotinylation reaction was quenched using 50 mm NH_4_Cl solution, after which cells were harvested and lysed in 1% NP‐40 lysis buffer (Beyotime). Biotinylated proteins were captured by overnight incubation with streptavidin agarose beads (YEASEN) at 4 °C and eluted using SDS‐PAGE sample buffer. Both total cell lysates (input) and biotinylated (cell surface) fractions were subsequently analyzed by Western blot.

### Receptor Recycling Assay

Biochemical assay. Surface proteins were labeled with cleavable Sulfo‐NHS‐SS‐biotin (0.5 mg mL^−1^; Apexbio, A8005) at 4 °C as described^[^ ^79^ ^]^ After CM‐PBS washing, cells were incubated at 37 °C for 30 min to permit endocytosis. Membrane trafficking was arrested by cooling cells to 4 °C, and residual surface biotin was removed by two sequential treatments with glutathione cleavage buffer. Cells were then incubated in serum‐free media containing 50 mm glutathione at 37 °C for various time periods to allow receptor recycling. After cooling to 4 °C, cells underwent two 15‐min treatments with ice‐cold glutathione cleavage buffer to remove biotin from recycled surface proteins. Remaining internalized biotinylated receptors were captured by streptavidin precipitation and analyzed by Western blot using anti‐CD36 and TREM2 antibodies, with the reduction in biotinylated proteins indicating the recycling rate.

Immunofluorescence assay. The receptor recycling assay was also performed using immunofluorescence as described previously.^[^ ^43^, ^69^ ^]^ Primary mouse microglia were seeded on PDL‐coated glass coverslips (Biosharp, BS‐20‐GJM) at 3 × 10⁵ cells well^−1^ and cultured in DMEM for 72 h, followed by 24‐h treatment with 1 µm oligomeric Aβ_1‐42_. Cells were blocked with 10% normal goat serum (37 °C, 15 min) and then incubated with anti‐CD36 and anti‐TREM2 antibodies (1:100 in 1% goat serum/DMEM) at 37 °C for 1 h. After acid stripping with ice‐cold DMEM (pH 2.0), cells were cultured in 10% goat serum/DMEM ± UA (100 µm) for 1 h. Cells were then labeled with Alexa Fluor 594‐conjugated secondary antibodies (1:1000 in 1% goat serum/DMEM) at 37 °C for 1 h, followed by acid washing to remove unbound antibodies. After fixation, nuclei were counterstained with Hoechst (Beyotime, 10 min). Recycled receptors were visualized using a NIKON AIR Plus confocal microscope and quantified as area intensity per cell using ImageJ software (NIH). The experimental scheme is shown in Figure S8F (Supporting Information).

### Statistical Analysis

All data are presented as means ± SEM, with in vitro experiments performed in triplicate. Statistical analyses were conducted using SPSS (v26.0), R software (v4.2.1) and GraphPad Prism (v8.02). Parametric or nonparametric tests were selected based on normality testing. Statistical significance was determined using two‐tailed Student's *t*‐test, Mann–Whitney tests, one‐way/two‐way ANOVA, Kruskal–Wallis tests, or Spearman's correlation analysis as appropriate. For baseline metabolite comparisons involving multiple testing, the Benjamini–Hochberg false discovery rate (FDR) procedure is applied with a prespecified FDR control level of 10% (*q* < 0.10) to correct for Type I error inflation. Multiple linear regression models were used to explore the association between baseline SUA and cognitive decline, as measured by CDR progression, adjusting for key covariates: sex, *APOE4* status, age, education, creatinine, blood urea nitrogen (BUN), diabetes, hypertension, hyperlipidemia, heart disease, stroke, smoking, and body mass index (BMI). Results were considered statistically significant at *P* < 0.05, except for FDR‐adjusted metabolite comparisons where significance was determined at *q* < 0.10. All experiments were conducted and analyzed by investigators blinded to experimental conditions, with sample sizes detailed in figure legends. No data were excluded from the analyses.

## Conflict of Interest

The authors declare no competing interests.

## Author Contributions

D.X. and Q.Z. contributed equally to this work. D.X., Q.Z., J.L., X.W., and J.C. designed the research. D.X., Q.Z., J.L., B.C., W.Q., X.Y., S.C., and S.H. performed the research. D.X., Q.Z., Z.C., W.Y., Y.X., and J.C. analyzed the data. S.F. and H.Z. contributed new reagents and analytic tools. D.X. and Q.Z. wrote the paper. T.Y., H.K., X.W., and J.C. reviewed the manuscript.

## Supporting information



Supporting Information

Supporting Information

## Data Availability

All data are available in the main text or the supplementary materials. The RNA‐seq data have been deposited into the CNGB Sequence Archive (CNSA) of China National GeneBank DataBase (CNGBdb) with the accession number CNP0006850.
