# Supplementary material for: Uric Acid Functions as an Endogenous Modulator of Microglial Function and Amyloid Clearance in Alzheimer's Disease
Source: Adv Sci (Weinh). 2025 Oct 6;12(48):e10270. doi: 10.1002/advs.202510270 (PMC12752628; doi:10.1002/advs.202510270)
Supplement: Supplementary file 1 — Supporting Information [file ADVS-12-e10270-s002.docx]

Supporting Information

Uric Acid Functions as an Endogenous Modulator of Microglial Function and Amyloid Clearance in Alzheimer’s Disease

De Xie, Qiuyang Zheng, Jiaming Lv, Qian Zhang, Zhiwei Cui, Shuai Huang, Wei Yu, Binyang Chen, Wanling Que, Shanpan Fu, Yuemei Xi, Jiayu Chen, Xueling Ye, Shuyi Chen, Hairong Zhao, Tetsuya Yamamoto, Hidenori Koyama, Xin Wang*, Jidong Cheng*

**This file includes:**

Figures S1 to S9

Tables S1 to S4

Supplementary Figures


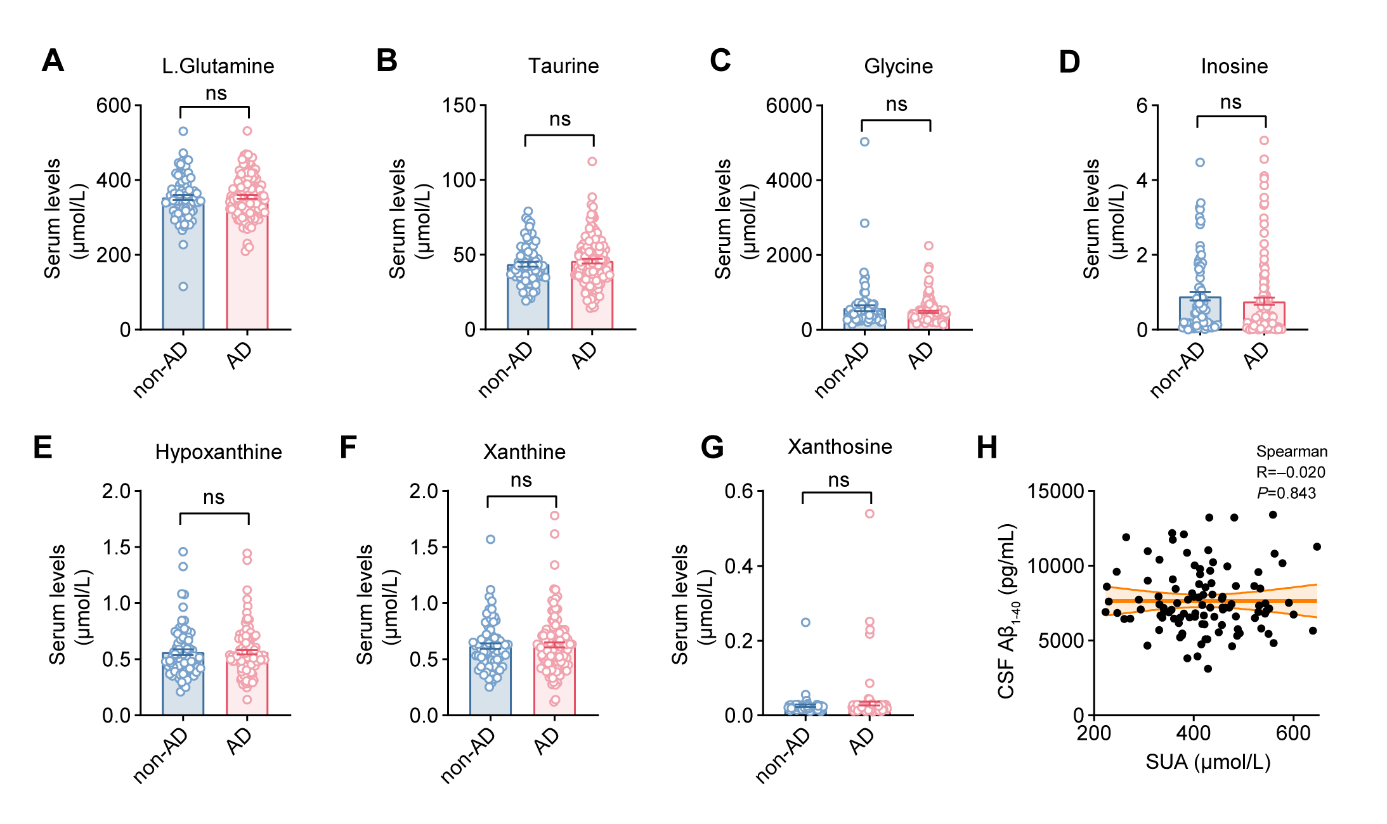


**Figure S1.** Analysis of purine metabolite profiles in AD patients. (A–G) Quantitative analysis of plasma purine metabolism in control subjects (non-AD; n = 76) and AD patients (n = 124), including L-glutamine (A), taurine (B), glycine (C), inosine (D), hypoxanthine (E), xanthine (F), and xanthosine (G). (H) Correlation analysis between baseline SUA and CSF Aβ_1-42_ levels in non-AD (n = 42) and AD subjects (n = 64). Data are presented as mean ± SEM. *P* values in (A–G) were determined by two-tailed Mann-Whitney test and adjusted using the Benjamini–Hochberg false discovery rate (FDR, *q* < 0.10); *P* values in (H) were determined by Spearman’s correlation analysis. ns, not significant.


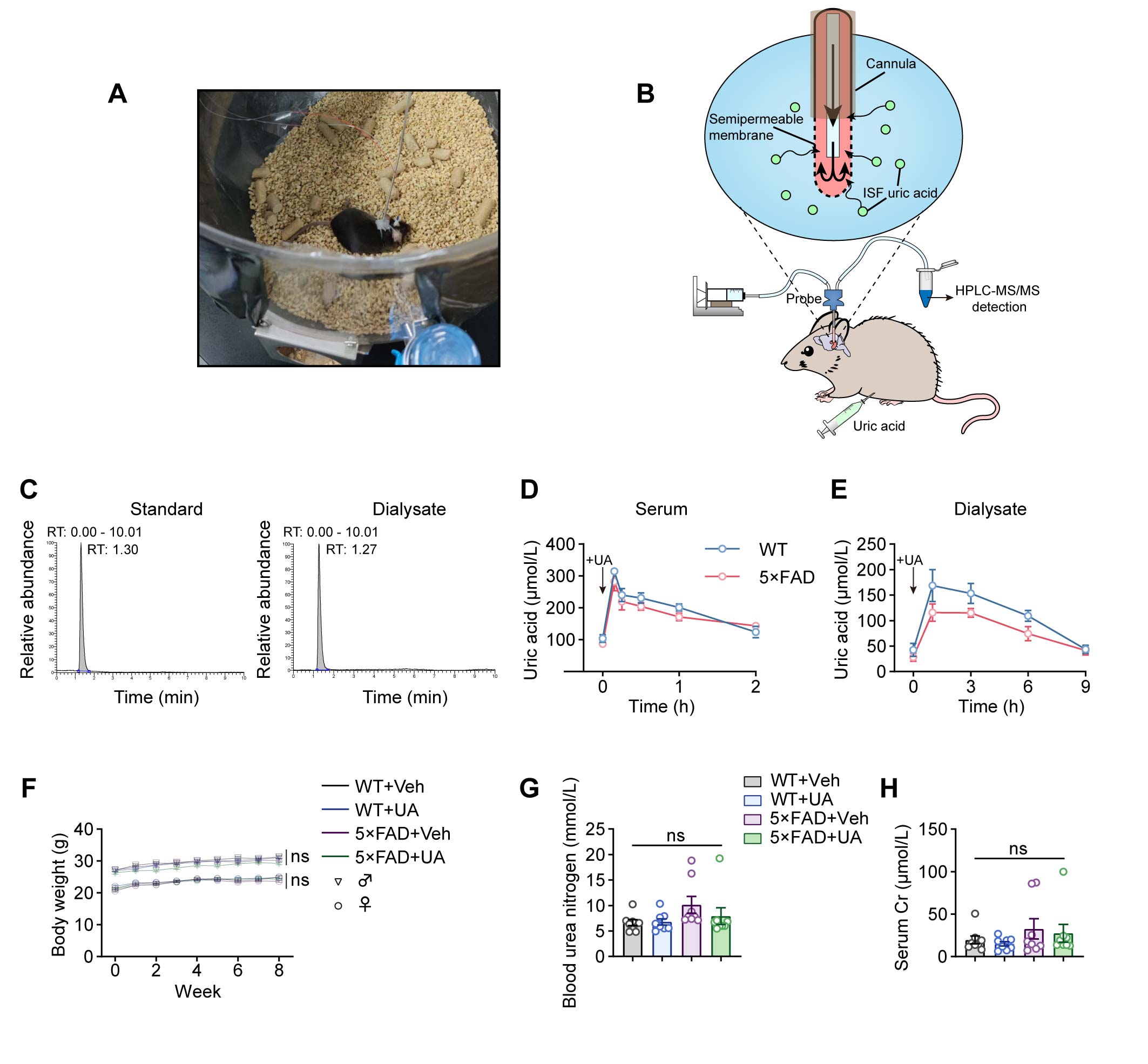
 **Figure S2.** *In vivo* microdialysis quantification of UA in interstitial fluid. (A) Experimental setup for awake, freely moving mice with implanted microdialysis probes, featuring a counterbalanced tethering system to minimize mechanical stress on the implant. (B) Schematic representative of *in vivo* microdialysis system for interstitial fluid (ISF) UA sampling. UA diffused across a 6 kDa molecular weight cut-off (MWCO) membrane according to concentration gradient. Dialysate collection performed over 12 h at a controlled flow rate. (C) High-performance liquid chromatography-mass spectrometry (HPLC-MS) chromatograms of UA standards and dialysate samples. UA peak identified in negative electrospray ionization (ESI) model (m/z: 167.0199, retention time: 1.30 min); peak integration performed using Xcalibur 4.1 software. (D and E) Temporal profiles of UA concentration in serum and microdialysates following single intraperitoneal UA injection (200 mg/kg) in 4-month-old WT and 5×FAD mice (n = 3 mice per group). UA recovery rate at 1.2 μL/min flow rate: 2.53 ± 0.93% (mean ± SEM). Data are presented as mean ± SEM. *P* values were determined by two-way ANOVA followed by Sidak’s multiple comparisons test in (D and E). ns, not significant.


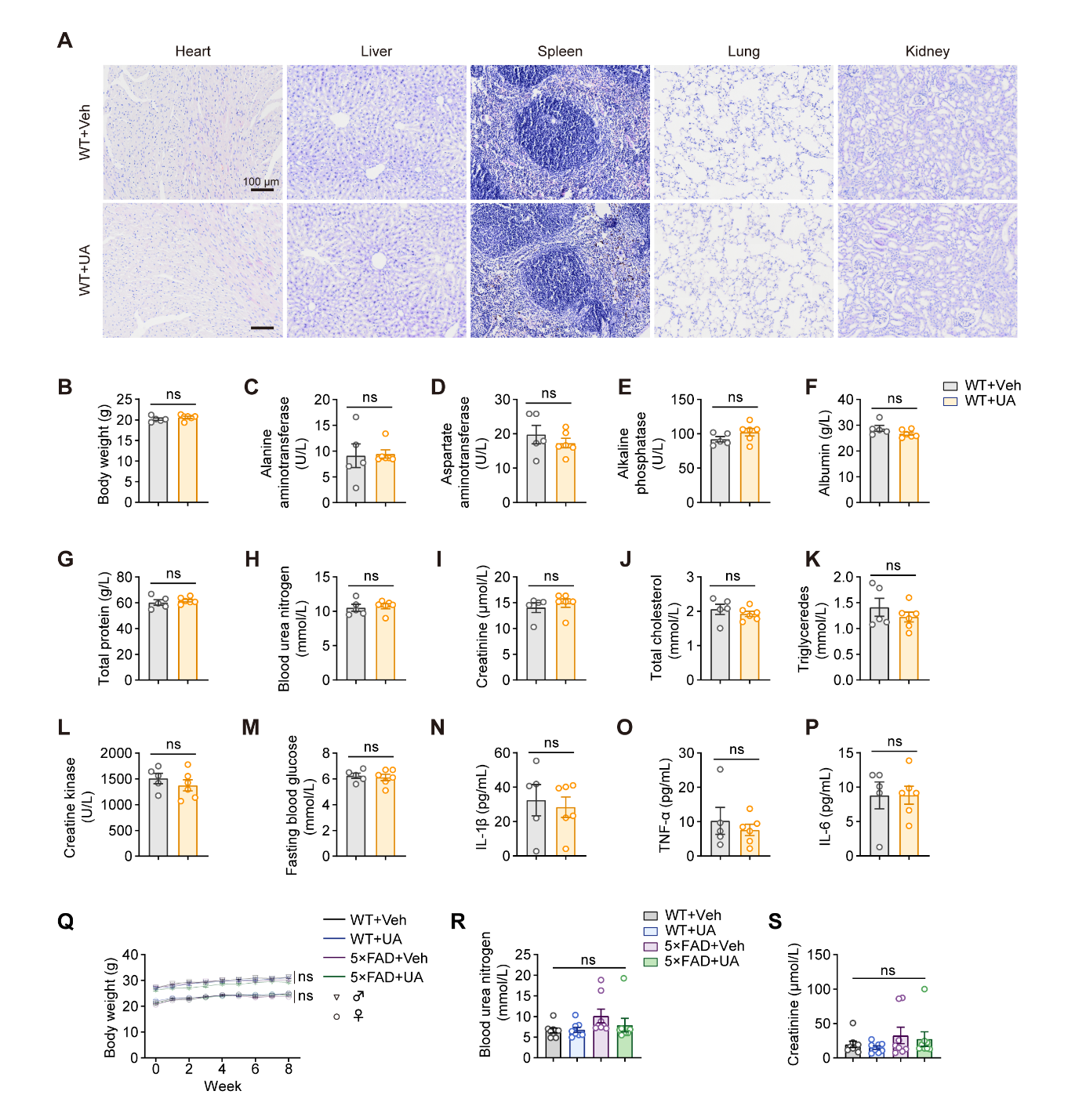


**Figure S3.** UA treatment does not cause adverse effects in mice. (A) H&E staining of vital organs (heart, liver, spleen, lung, and kidney) from WT mice after 2-week intraperitoneal injection of vehicle or UA. Scale bar, 100 μm. (B-P) Body weight and serum biochemistry profiles after 2-week treatment (n = 5 in WT+vehicle; n = 6 in WT+UA). (Q) Longitudinal body weight measurements during 2-month vehicle (Veh) or UA treatment in WT and 5×FAD mice (n = 10 mice per group). (R) Blood urea nitrogen and (S) serum creatinine concentrations following indicated treatments (n = 8 mice per group). Data are presented as mean ± SEM. *P* values were determined by two-tailed unpaired Student’s *t*-test in (B, D-G, J-P), two-tailed Mann-Whitney test in (C, H and I), two-way ANOVA followed by Sidak’s multiple comparisons test in (Q) and Kruskal-Wallis test followed by Dunn’s *post hoc* analysis in (R and S). ns, not significant.


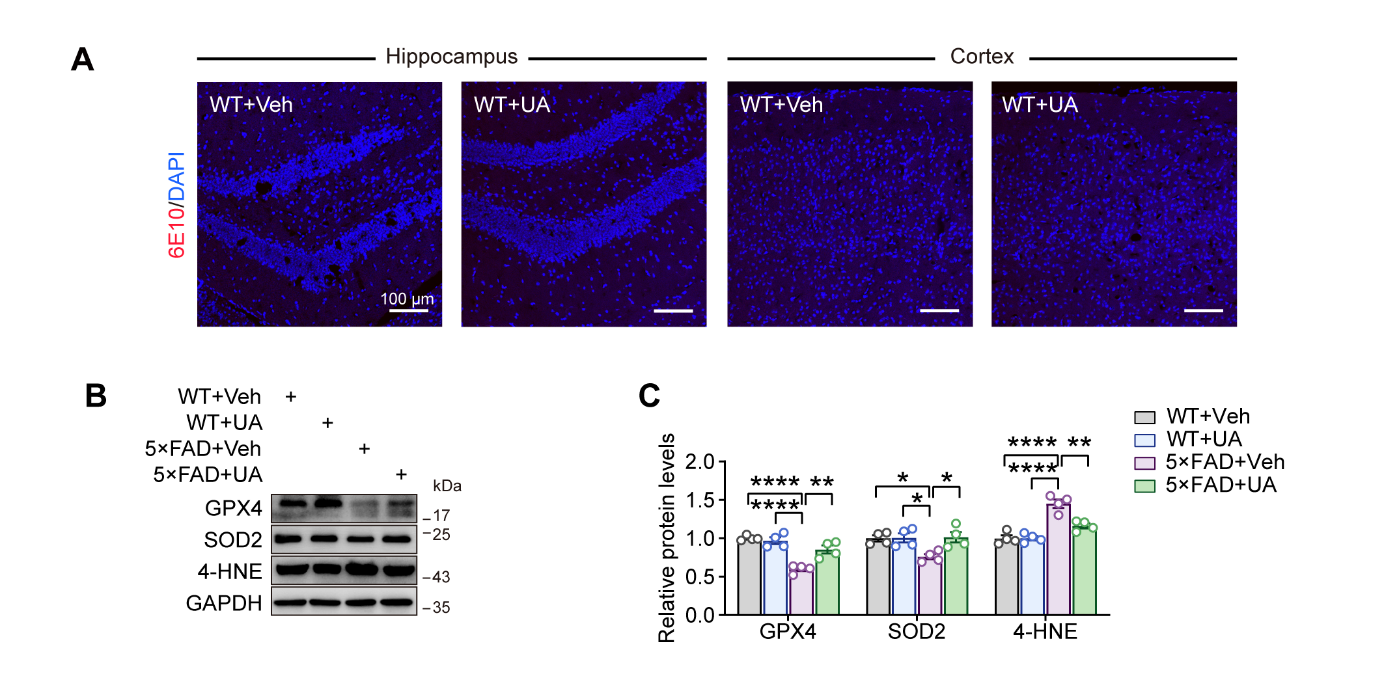


**Figure S4.** UA alleviates oxidative stress in AD mouse brain. (A) Immunofluorescence staining of amyloid plaque (6E10, red) in hippocampal and cortical sections from WT+Veh and WT+UA mice. Scale bar, 100 μm. (B and C) Western blot analysis of GPX4, SOD2, and 4-HNE in hippocampal tissue (n = 4 mice per group). Data are presented as mean ± SEM. *P* values were determined by one-way ANOVA followed by Tukey’s *post hoc* analysis in (C). **P* < 0.05; ***P* < 0.01; *****P* < 0.0001.

**
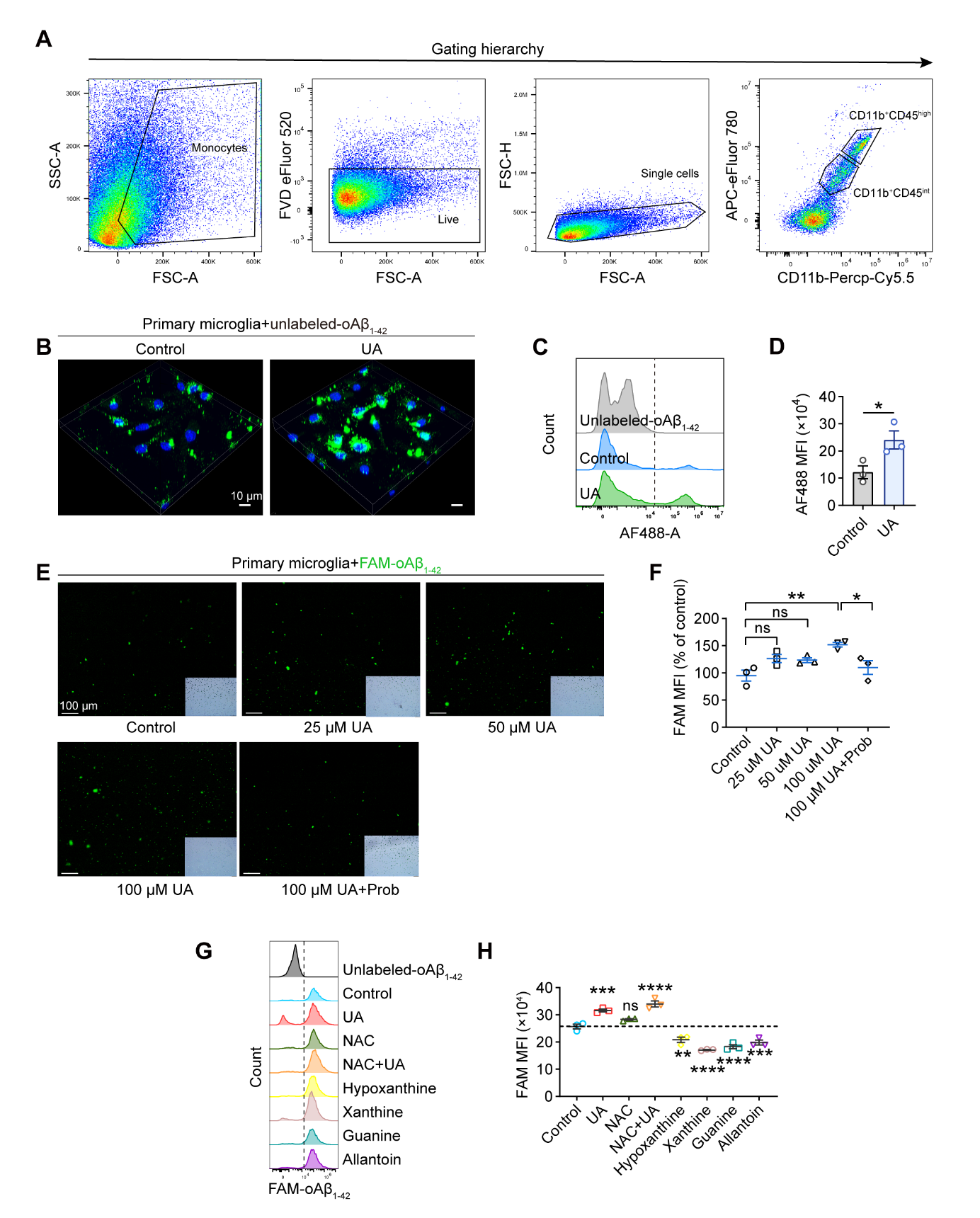
**

**Figure S5.** UA enhances microglial phagocytosis of Aβ. (A) Flow cytometry gating strategy for microglial isolation employed sequential gating using forward/side scatter (FSC-A/SSC-A) for whole cells, fixable viability dye (FVD) eFluor 520 for live cells, FSC-A/FSC-H for singlets, and CD45^+^ (APC-eFluor 780)/CD11b^+^ (Percp-Cy5.5) for microglia/macrophage population. (B) Representative confocal z-stack images and (C) flow cytometry histograms of primary microglia exposure to unlabeled-oAβ_1-42_ (1 μM, 3 h) following vehicle or UA pretreatment (100 μM, 12 h). Aβ visualized using Alexa Fluor (AF) 488-conjugated secondary antibody (green). (D) Quantification of MFI from (C) (n = 3 independent experiments). (E) Comparative analysis of primary microglial responses across indicated distinct treatment with or without probenecid pretreatment (100 μM, 2 h). Scale bar, 100 μm. (F) Quantification of FAM-oAβ_1-42_ uptake from (E) using fluorescence microscopy (n = 3 independent experiments). (G) Flow cytometry analysis of FAM-oAβ_1-42_ (1 μM, 3 h) uptake in BV2 microglia following 12-h pretreatment with vehicle, 100 μM UA, 10 μM NAC, 10 μM NAC+100 μM UA, 10 μM hypoxanthine, 10 μM xanthine, 10 μM guanine or 100 μM allantoin. (H) Quantification of MFI from (G) (n = 3 independent experiments). Data are presented as mean ± SEM. *P* values were determined by two-tailed unpaired Student’s *t*-test in (D) and one-way ANOVA followed by Tukey’s *post hoc* analysis in (F and H). ns, not significant; **P* < 0.05; ***P* < 0.01; ****P* < 0.001; *****P* < 0.0001.

**
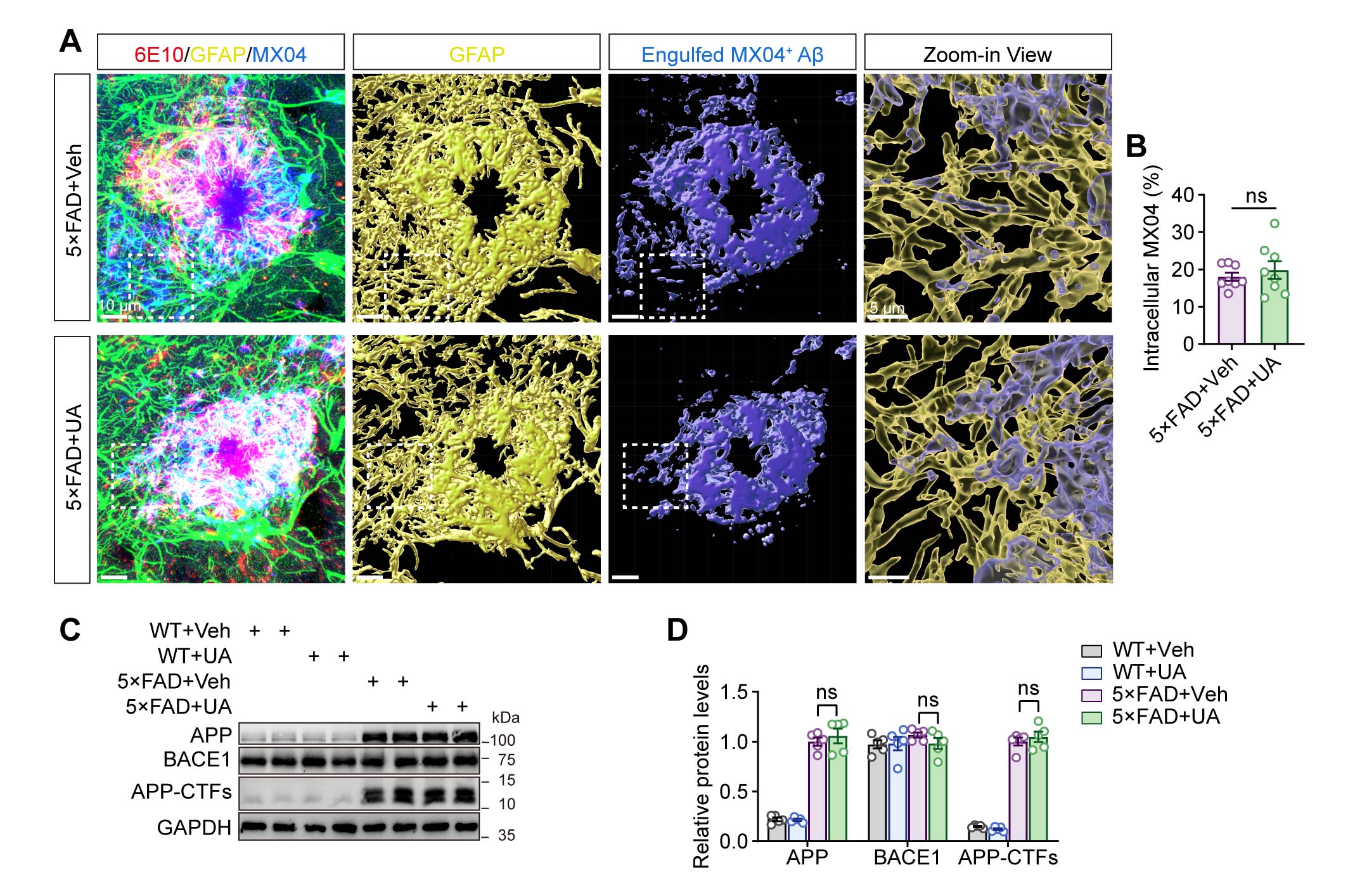
Figure S6.** UA does not affect astrocytic Aβ phagocytosis or APP processing. (A) Representative Imaris-based 3D reconstruction of amyloid plaque (MX04, blue; 6E10, red) and astrocytes (GFAP, yellow). Scale bar, 10 μm; 5 μm in zoom-in view. (B) Quantification of MX04-positive plaques internalization by astrocytes. n = 38-44 plaques from 8 volumetric images of 8 mice per group. (C and D) Western blot analysis of APP, BACE1 and APP-CTFs in hippocampal tissue (n = 5 mice per group). Data are presented as mean ± SEM. *P* values were determined by two-tailed unpaired Student’s *t*-test in (B), one-way ANOVA followed by Tukey’s *post hoc* analysis in (D BACE1), and Kruskal-Wallis test followed by Dunn’s *post hoc* analysis in (D APP and APP-CTFs). ns, not significant.


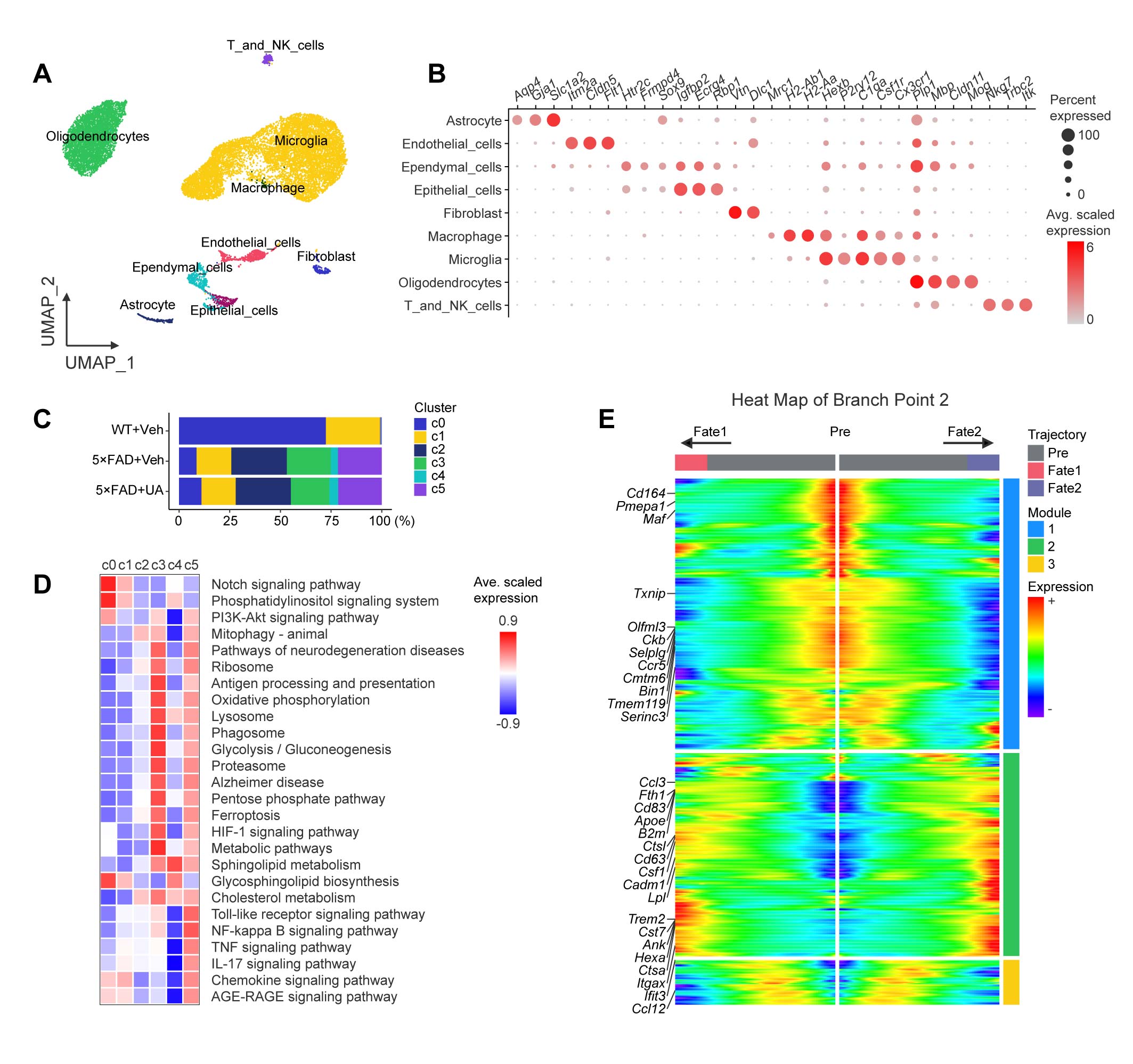
 **Figure S7.** Single-cell transcriptional profiling reveals microglial heterogeneity in UA-treated 5×FAD mouse hippocampus. (A) Uniform manifold approximation and projection (UMAP) dimensional reduction plot revealing cellular heterogeneity and major cell types clusters in hippocampal tissues from WT+Veh, 5×FAD+Veh and 5×FAD+UA mice at 6.5 months of age. (B) Bubble plots visualization of cell type-specific marker gene expression patterns for clusters identified in (A). (C) Quantitative analysis of cell type distribution (cluster fractions) within total hippocampal cell population across indicated groups. (D) Heatmap representation of quantitative set analysis of gene expression (QuSAGE) results comparing gene set enrichment across six distinct microglial clusters. (E) Hierarchical clustering analysis heatmap of differentially expressed genes (DEGs) organized into three modules identified through pseudotime trajectory analysis at branch point 2.

**
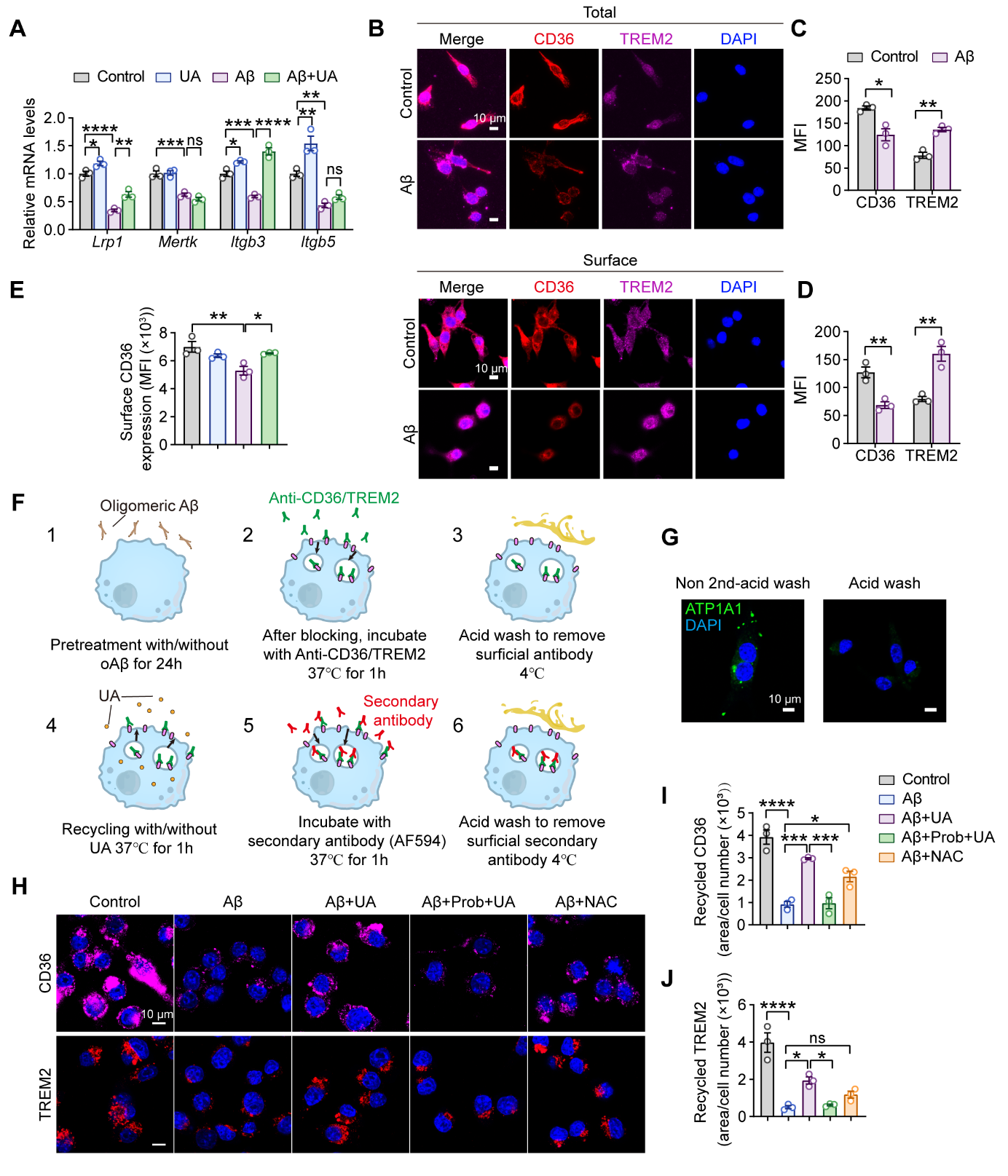
Figure S8.** UA promotes recycling of phagocytic receptors in microglia. (A) Quantitative RT-PCR analysis of Aβ phagocytosis-associated receptor transcripts in primary microglia following treatment with vehicle (control), UA, Aβ, or UA+Aβ (n = 3 independent experiments). (B) Representative confocal images and quantification of total (C) and surface (D) CD36 and TREM2 expression in primary microglia after Aβ treatment (n = 3 independent experiments). (E) Flow cytometry analysis of CD36 surface expression (n = 3 independent experiments). (F) Schematic representation of receptor recycling assay methodology. Sequential analysis (steps 1-6) of CD36 and TREM2 recycling in primary microglia, followed by DAPI nuclear staining and confocal microscopy. (G) Representative confocal images of the resident cell surface marker ATP1A1 with or without secondary acid wash in receptor recycling assay. (H) Representative confocal images showing receptor recycling of CD36 and TREM2 in BV2 microglia after 24-hour pretreatment with vehicle, 1 μM Aβ or 100 μM probenecid, followed by treatment with 100 μM UA or 10 μM NAC to assess recycling (scale bar, 10 μm). (I and G) Quantification of receptors recycling from (H) (n = 3 independent experiments). Data are presented as mean ± SEM. *P* values were determined by one-way ANOVA followed by Tukey’s *post hoc* analysis in (A, E, I and J) and two-tailed unpaired Student’s *t*-test in (C and D). ns, not significant; **P* < 0.05; ***P* < 0.01; ****P* < 0.001; *****P* < 0.0001.


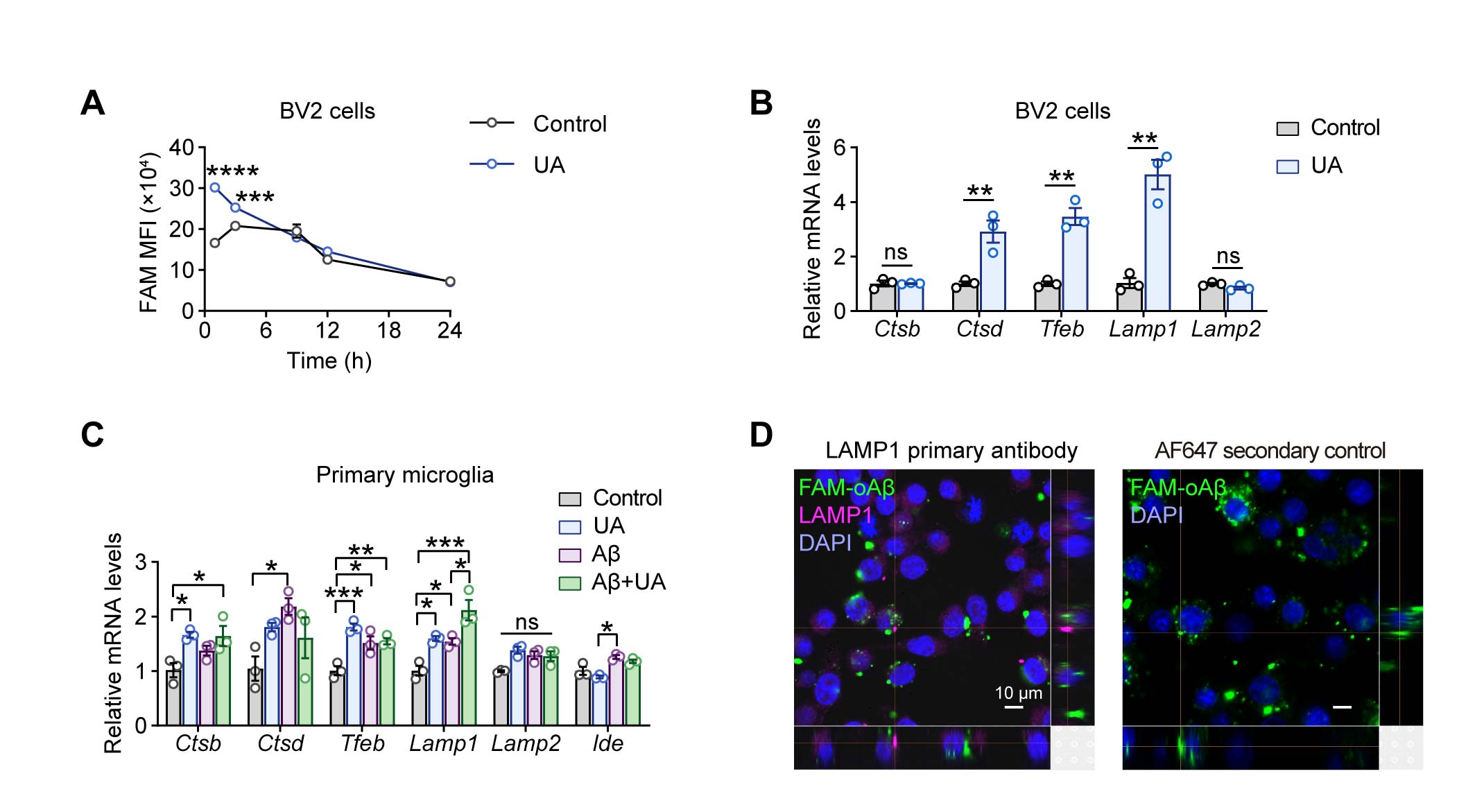
 **Figure S9.** UA enhances microglial clearance of Aβ. (A) Flow cytometry analysis of Aβ clearance kinetics in BV2 microglia following UA pretreatment (100 μM, 12 h) and subsequent challenge with oligomeric FAM-oAβ_1-42_ (1 μM) exposure at varying time points (n = 3 independent experiments). (B) Quantitative RT-PCR analysis of lysosome-associated gene expression in BV2 cells after UA treatment (100 μM, 12 h) (n = 3 independent experiments). (C) Quantitative RT-PCR analysis of Aβ-degrading enzyme transcripts in primary microglia following UA pretreatment (100 μM, 6 h) and subsequent challenge with oligomeric Aβ (1 μM, 3 h) (n = 3 biologically independent samples). (D) Representative confocal images visualized LAMP1-positive lysosomes with or without Alexa Fluor (AF) 647-conjugated secondary antibody following FAM-oAβ_1-42_ exposure (6 h). Scale bar, 10 μm. Data are presented as mean ± SEM. *P* values were determined by two-way ANOVA followed by Sidak’s multiple comparisons test in (A), two-tailed unpaired Student’s *t*-test in (B), one-way ANOVA followed by Tukey’s *post hoc* analysis test in (C: *Ctsb*, *Ctsd*, *Tfeb* and *Lamp1*), and Kruskal-Wallis test followed by Dunn’s *post hoc* analysis in (C: *Lamp2* and *Ide*). ns, not significant; **P* < 0.05; ***P* < 0.01; ****P* < 0.001; *****P* < 0.0001.

Supplementary Tables

Table S1. Demographic characteristics, CSF biomarkers and clinical parameters of study participants.

|  | non-AD | AD | *P* value |
| --- | --- | --- | --- |
| **Demographics** | | | |
| Number (%) | 76 (38%) | 124 (62%) |  |
| Age, years | 75.5 (4.7) | 74.5 (7.5) | 0.7215 |
| Female, N (%) | 30 (39.0%) | 43 (34.7%) | 0.5429 |
| Education, years | 16.4 (2.9) | 15.8 (3.1) | 0.2010 |
| *APOE4* carrier | 17 (22.3%) | 70 (56.5%) | < 0.0001 |
| **Cardiovascular risk factors** | | | |
| Diabetes | 8 (10.7%) | 13 (11.0%) | 0.9393 |
| Hypertension | 37 (48.7%) | 49 (39.5%) | 0.2037 |
| Hyperlipidemia | 19 (25.3%) | 42 (35.6%) | 0.1351 |
| Heart disease | 70 (92.1%) | 113 (91.1%) | 0.8101 |
| Stroke | 1 (1.3%) | 3 (2.4%) | 0.9834 |
| Smoking | 27 (40.9%) | 38 (36.5%) | 0.5677 |
| Body Mass Index (kg/m^2^) | 27.7 (0.5) | 25.7 (0.3) | < 0.001 |
| **Renal function** | | | |
| CR (mg/dL) | 0.9933 (0.03) | 0.9915 (0.02) | 0.9942 |
| BUN (mg/dL) | 19.48 (0.7) | 19.08 (0.5) | 0.9153 |
| **Baseline CSF biomarkers** | | | |
| Aβ_1-40_, pg/mL | 7883 (2292) | 7508 (1964) | 0.3751 |
| Aβ_1-42_, pg/mL | 1299 (600) | 808 (435) | < 0.0001 |
| **Cognition** | | | |
| Baseline MMSE | 29.1 (1.0) | 26.0 (2.6) | < 0.0001 |

CSF, cerebrospinal fluid; *APOE4*, apolipoprotein E4; CR, creatinine; BUN, blood urea nitrogen; MMSE, Mini-Mental State Examination. n (non-AD) = 76, n (AD) = 124. Data are presented as mean ± SEM. *P* values were determined by two-tailed Mann-Whitney test (Age, education, CSF Aβ_1-42_, MMSE, CR and BUN), two-sided Fisher’s exact test (Gender, *APOE4* carrier, diabetes, hypertension, hyperlipidemia, heart disease, stroke, and smoking), and two-tailed unpaired Student’s *t*-test (CSF Aβ_1-40_ and body mass index).

**Table S2. Association between baseline serum UA levels and annual change in CDR scores.**

|  | Model 1 | | | Model 2 | |
| --- | --- | --- | --- | --- | --- |
|  | β (95% CI) | *P* value | | β (95% CI) | *P* value |
| SUA, +1 mg/dL | –0.223  (–0.052, -0.001) | | 0.004 | –0.203  (–0.053, –0.011) | 0.043 |

Data are presented using multiple linear regression models. Model 1: adjusted for sex and *APOE4* status*.* Model 2: adjusted for sex, *APOE4* status, age, education, CR, BUN, diabetes, hypertension, hyperlipidemia, heart disease, stroke, smoking, and body mass index.

β: regression coefficient; CI: confidence interval; SUA: serum uric acid; CR: Creatinine; BUN: blood urea nitrogen.

Table S3. Primary and secondary antibodies used in this study.

| **Antibodies** | **Source/**  **catalog no.** | **Western blot** | **Immune fluorescence/Flow cytometry** |
| --- | --- | --- | --- |
| Mouse monoclonal anti-β-amyloid, 1-16 antibody (clone 6E10) | Biolegend, 803001 | - | 1:400 |
| Rabbit polyclonal anti- LAMP1 | Abcam, 24170 | - | 1:200 |
| Rabbit monoclonal anti-Iba1 | Abcam, 178847 | - | 1:100 |
| Rabbit polyclonal anti-GFAP | Abcam, 7260 | - | 1:200 |
| Mouse monoclonal anti-APP | Millipore, MAB348 | 1:1000 | - |
| Rabbit monoclonal anti-BACE1 | CST, 5606 | 1:1000 | - |
| Rabbit monoclonal anti-APP-CTFs | Abcam, 32136 | 1:1000 | - |
| Rabbit polyclonal anti-CD36 | Novusbio, NB400-144 | 1:1000 | 1:100 |
| Rat monoclonal anti-TREM2 | R&D systems, 17291 | - | 1:100 |
| Rabbit monoclonal anti-TREM2 | Proteintech, 27599 | 1:1000 | - |
| Mouse monoclonal anti- Na^+^/K^+^-ATPase | Sigma, 05369 | 1:1000 | 1:100 |
| Mouse monoclonal anti-GAPDH | ABclonal AC002 | 1:5000 | - |
| Mouse monoclonal anti-β-actin | ABclonal AC004 | 1:5000 | - |
| Rabbit monoclonal anti-GPX4 | ABclonal A11243 | 1:1000 | - |
| Rabbit monoclonal anti-SOD2 | Proteintech, 24127 | 1:1000 | - |
| Rabbit monoclonal anti-4-HNE | ABclonal A26085 | 1:1000 | - |
| Goat anti-mouse IgG | Bioss, bs-0296G | 1:1000 | - |
| Goat anti-rabbit IgG | Bioss, bs-0295G | 1:1000 | - |
| Goat anti-rat IgG | Abclonal, AS028 | 1:1000 | - |
| Alexa Fluor^®^ 488-conjugated anti-rabbit IgG | Abcam, ab150077 | - | 1:500 |
| Alexa Fluor^®^ 488-conjugated anti-mouse IgG | CST, 4408 | - | 1:500 |
| Alexa Fluor® 594-conjugated anti-mouse IgG | Invitrigen, A11005 | - | 1:500 |
| Alexa Fluor® 647-conjugated anti-rabbit IgG | Invitrogen, A21245 | - | 1:500 |
| Purified rat anti-mouse CD16/CD32 | BD, 553141 | - | 1:100 |
| Fixable viability dye eFluor™ 520 | Invitrogen, 65-0867-18 | - | 1:1000 |
| APC-eFluor™ 780 CD45 monoclonal antibody | Invitrogen, 47-0451-82 | - | 1:100 |
| PerCP-Cy™5.5 rat anti-CD11b | BD, 550993 | - | 1:100 |

Table S4. Quantitative RT-PCR primer information.

| **Target gene** | **Forward sequence** | **Reverse sequence** |
| --- | --- | --- |
| *Cd36* | GAACCACTGCTTTCAAAAACTGG | TGCTGTTCTTTGCCACGTCA |
| *Msr1* | TGAACGAGAGGATGCTGACTG | GGAGGGGCCATTTTTAGTGC |
| *Tlr4* | AGGCACATGCTCTAGCACTAA | AGGCTCCCCAGTTTAACTCTG |
| *Trem2* | CTGGAACCGTCACCATCACTC | CGAAACTCGATGACTCCTCGG |
| *Abca7* | GCCAGTATGGAATCCCTGAA | ATGGAGACACCAGGAACCAG |
| *Lrp1* | CCACTATGGATGCCCCTAAAAC | GCAATCTCTTTCACCGTCACA |
| *Mertk* | TGCGTTTAATCACACCATTGGA | TGCCCCGAGCAATTCCTTTC |
| *Avb3* | CCACACGAGGCGTGAACTC | CTTCAGGTTACATCGGGGTGA |
| *Avb5* | GAAGTGCCACCTCGTGTGAA | GGACCGTGGATTGCCAAAGT |
| *Tfeb* | GCTCCAACCCCGAGAAAGAG | CAGCGTGTTAGGCATCTGC |
| *Arsa* | CTGGGGACCCTCTTTTTGGC | AACTGGGGTGCCCATAGGA |
| *Arsb* | CCTCCGCATGTGGTCTTCG | CGGCTGCACGTAGTAGTTGTC |
| *Atp6v1h* | CCAAGATGGACATTCGAGGTG | CACTTTGTTGGCACGAACTTC |
| *Atp6v0e1* | GCATACCACGGCCTTACTGT | TGATAACTCCCCGGTTAGGAC |
| *Clcn7* | GACAACAGCGAGAATCAGCTC | CCAATGAGGGCACAGATAACC |
| *Ctsa* | CAGCCCTCTTTCCGGCAATA | TTTGGGTCGTTCTGCGACTC |
| *Ctsb* | GAAGCCATTTCTGACCGAAC | CACCTGAAACCAGGCCTTT |
| *Ctsd* | CATGCAGTCATCGCCTAAGA | AGGGGACCCACAGGTTAGAG |
| *Ctsf* | TGACCACCTATAACCGGACTT | TGGTGATCCCATACTGAGCTG |
| *Galns* | TCATGGACGATATGGGGTGG | AGATGGTGAGCACAAAGGGTT |
| *Gba* | GCCAGGCTCATCGGATTCTTC | GAGTGCTCTCGTAACGGCT |
| *Gla* | GCTCCCGAGAGGGATTCAAAG | CTGTGGACGTAATTTGCGAGG |
| *Gns* | GGCATGACGCCACTGAAGAA | GGGCACATAGGCGCTAGAG |
| *Hexa* | TGGCCCCAGTACATCCAAAC | GGTTACGGTAGCGTCGAAAGG |
| *Mcoln1* | GCTGGGTTACTCTGATGGGTC | CCACCACGGACATAGGCATAC |
| *Naglu* | ACCGCTATTACCAGAATGTGTG | GTGTGCAAGTTACCCATGCG |
| *Neu1* | GGACCGCTGAGCTATTGGG | CGGGATGCGGAAAGTGTCTA |
| *Psap* | CCTGTCCAAGACCCGAAGAC | AAGGAAGGGATTTCGCTGTGG |
| *Scpep1* | CTGCTGCTCCTATCGTTCTTAC | TCGGACAGTCACATAATCCCATA |
| *Sgsh* | CGTGCTACTGATAGTTGCGGA | GTTACGGAAGATAAGGCTGTGG |
| *Tmem55b* | ACTCACCCTTGACTAGCCC | TCCACGTTGATCGGAGACTGA |
| *Tpp1* | GAGTCTCACTTTTGCGCTGAA | CTCCAGGGTTAGGTACTTTCCA |
| *Tfeb* | GCTCCAACCCCGAGAAAGAG | CAGCGTGTTAGGCATCTGC |
| *Lamp1* | TAGTGCCCACATTCAGCATCT | TCCTGCCAATGAGGTAGGCAATGA |
| *Lamp2* | CCATTGGATGTCATCTTTAAGTGC | GTTGAAAGCTGAGCCATTAG |
| *Ide* | AATCCGGCCATCCAGAGAATA | GGGTCTGACAGTGAACCTATGT |
