# Supplementary material for: Uric Acid Functions as an Endogenous Modulator of Microglial Function and Amyloid Clearance in Alzheimer's Disease
Source: Adv Sci (Weinh). 2025 Oct 6;12(48):e10270. doi: 10.1002/advs.202510270 (PMC12752628; doi:10.1002/advs.202510270)

Original blots Fig. 6D

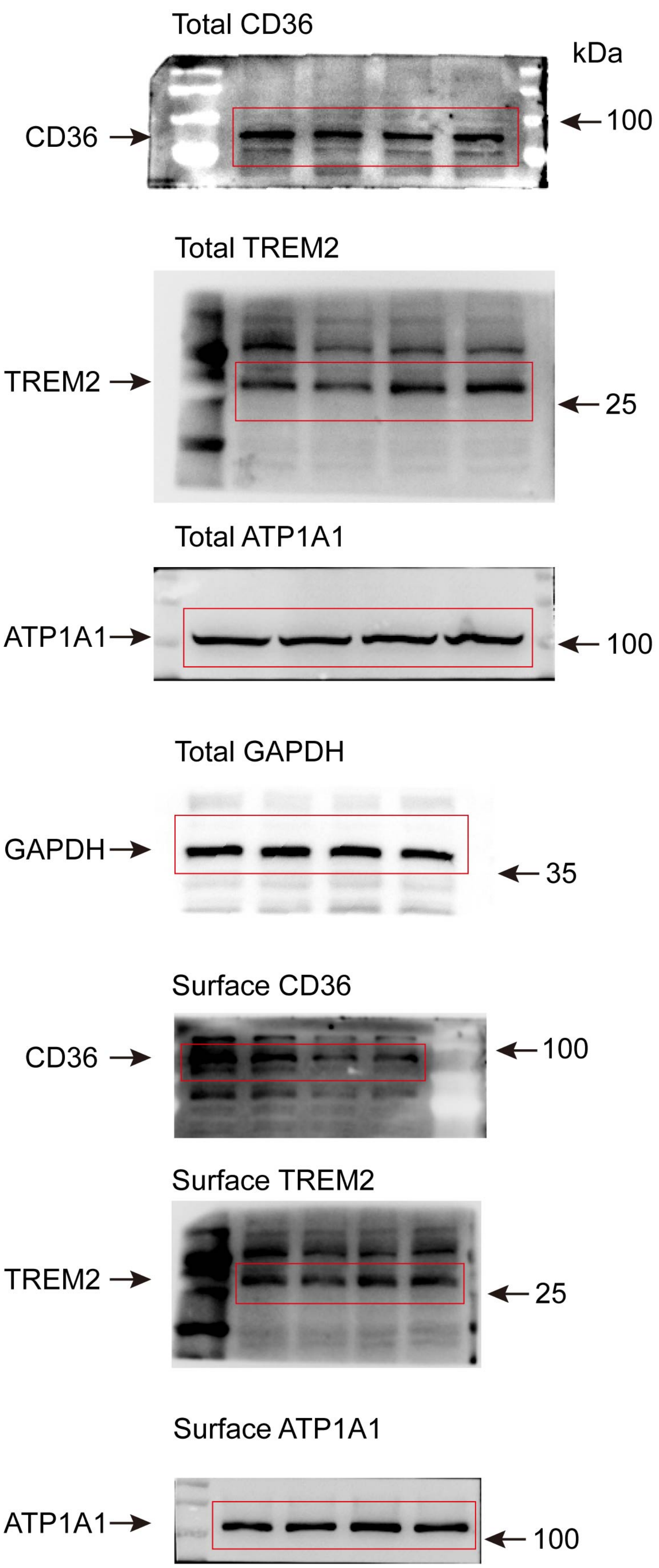

Original blots Fig. 6H

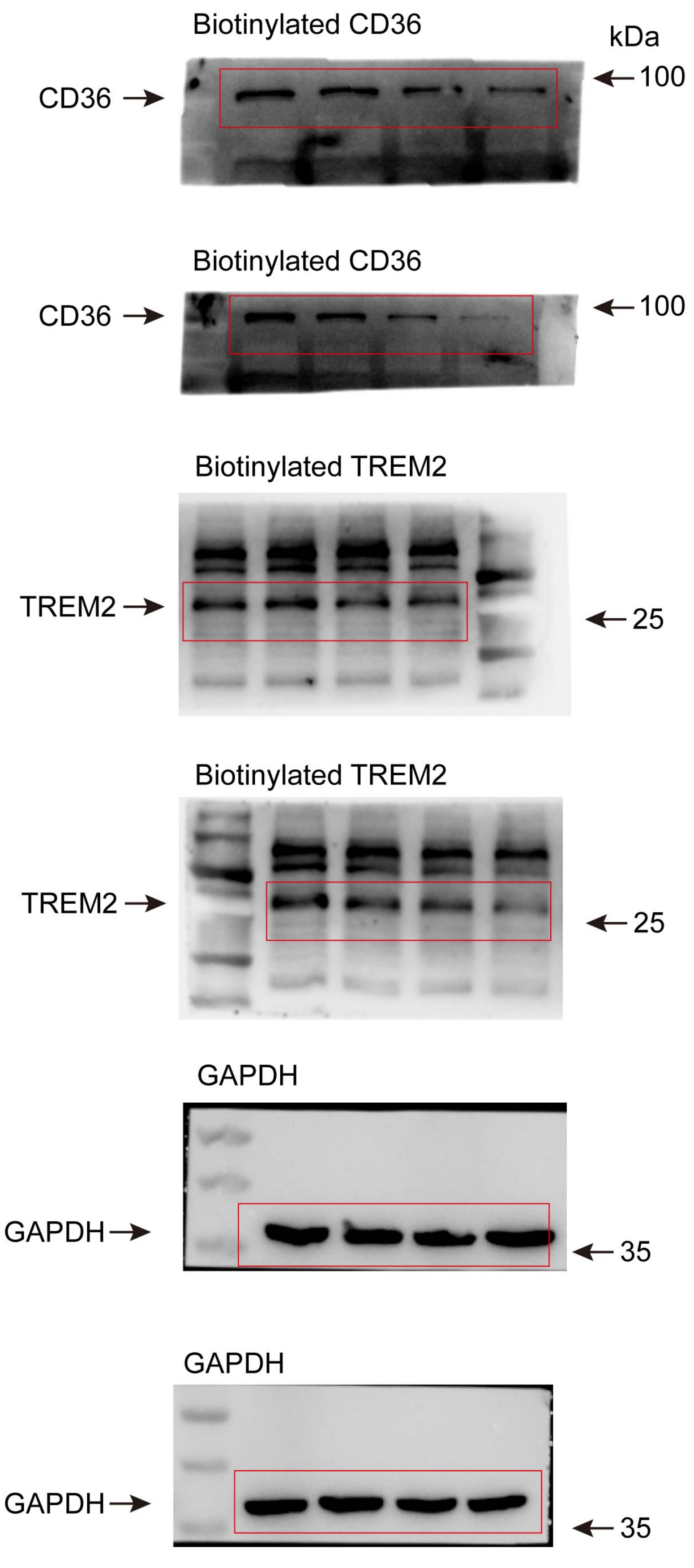

Original blots Supporting information, Fig. S4B

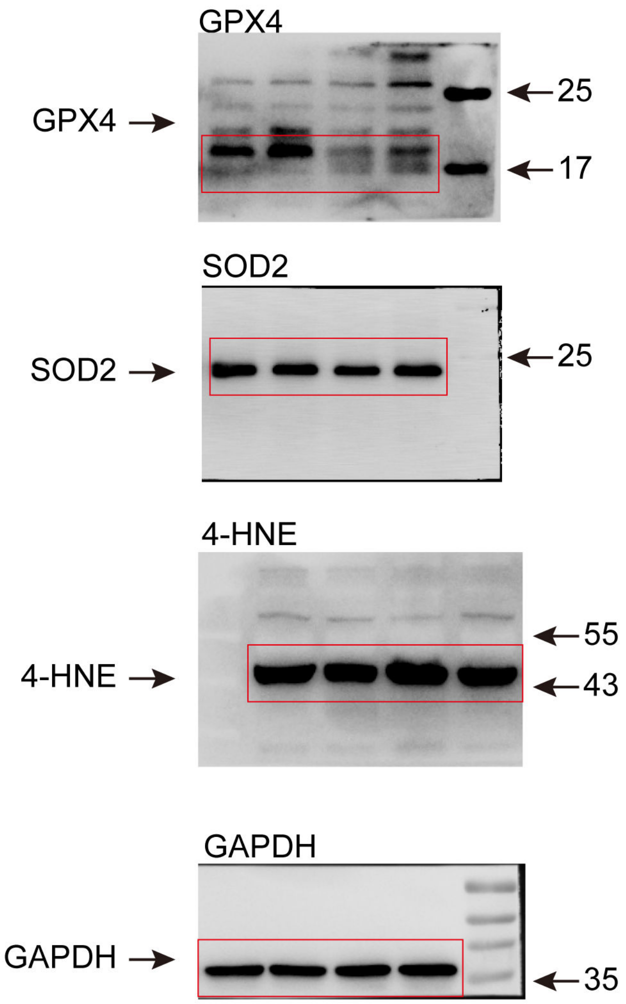

Original blots Supporting information, Fig. S6C

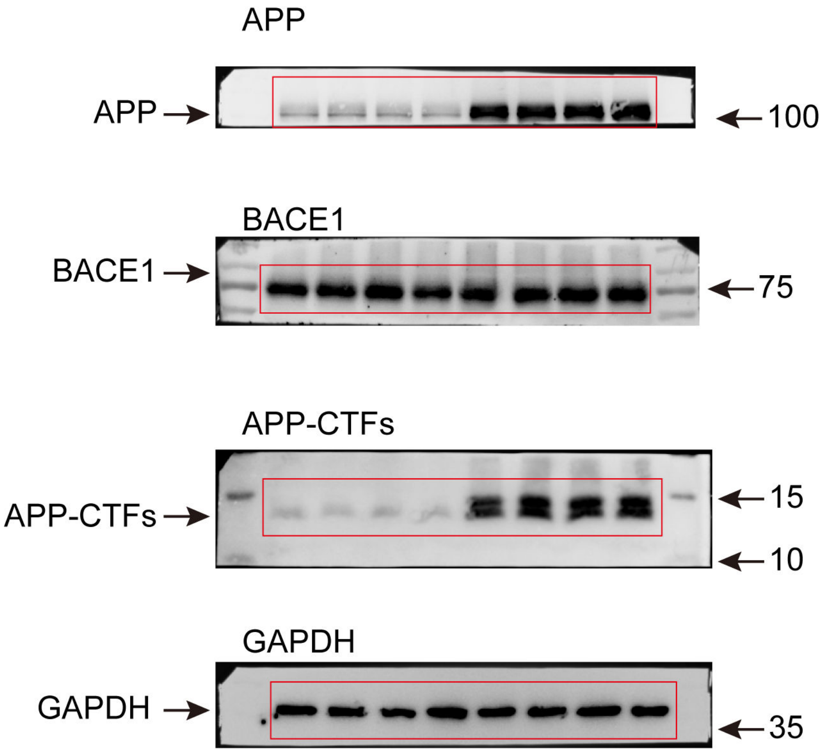

Supplement: Supplementary file 2 — Supporting Information [file ADVS-12-e10270-s001.zip › Original blots.pdf]
